# DaXi—high-resolution, large imaging volume and multi-view single-objective light-sheet microscopy

**DOI:** 10.1038/s41592-022-01417-2

**Published:** 2022-03-21

**Authors:** Bin Yang, Merlin Lange, Alfred Millett-Sikking, Xiang Zhao, Jordão Bragantini, Shruthi VijayKumar, Mason Kamb, Rafael Gómez-Sjöberg, Ahmet Can Solak, Wanpeng Wang, Hirofumi Kobayashi, Matthew N. McCarroll, Lachlan W. Whitehead, Reto P. Fiolka, Thomas B. Kornberg, Andrew G. York, Loic A. Royer

**Affiliations:** 1 Chan Zuckerberg Biohub, San Francisco, CA USA; 2 Calico Life Sciences LLC, South San Francisco, CA USA; 3 Cardiovascular Research Institute, University of California, San Francisco (UCSF), San Francisco, CA USA; 4 Department of Pharmaceutical Chemistry, University of California, San Francisco (UCSF), San Francisco, CA USA; 5 The Walter and Eliza Hall Institute of Medical Research, Parkville, Victoria Australia; 6 Department of Medical Biology, The University of Melbourne, Parkville, Victoria Australia; 7 Department of Cell Biology, University of Texas Southwestern Medical Center, Dallas, TX USA; 8 Lyda Hill Department of Bioinformatics, University of Texas Southwestern Medical Center, Dallas, TX USA

**Keywords:** Light-sheet microscopy, Embryogenesis

## Abstract

The promise of single-objective light-sheet microscopy is to combine the convenience of standard single-objective microscopes with the speed, coverage, resolution and gentleness of light-sheet microscopes. We present DaXi, a single-objective light-sheet microscope design based on oblique plane illumination that achieves: (1) a wider field of view and high-resolution imaging via a custom remote focusing objective; (2) fast volumetric imaging over larger volumes without compromising image quality or necessitating tiled acquisition; (3) fuller image coverage for large samples via multi-view imaging and (4) higher throughput multi-well imaging via remote coverslip placement. Our instrument achieves a resolution of 450 nm laterally and 2 μm axially over an imaging volume of 3,000 × 800 × 300 μm. We demonstrate the speed, field of view, resolution and versatility of our instrument by imaging various systems, including *Drosophila* egg chamber development, zebrafish whole-brain activity and zebrafish embryonic development – up to nine embryos at a time.

## Abstract (web summary)

The DaXi single-objective light-sheet microscope achieves fast, high-quality imaging of large volumes. DaXi’s design allows increased scanning range without sacrificing imaging speed or quality, multiview imaging and versatile sample mounting.

## Main

In recent years, light-sheet microscopy has become an essential imaging method for biology^1,2^. It has had a particular impact on developmental biology, allowing the first in toto volumetric reconstructions of embryonic development of model organisms such as *Drosophila*, zebrafish and mouse^1–3^. The ability to image whole developmental arcs and to follow hundreds of thousands of cells in space and time has shown the potential of light-sheet microscopy in answering long-standing questions^3,4^.

However, a major limitation in light-sheet microscopy is the requirement of complex multi-objective configurations that complicate sample mounting. Recent developments have attempted to improve the sample mounting ergonomics with open-top imaging systems^5–8^ or by alleviating the necessity for orthogonal objectives^9^. Among these methods, the oblique plane microscope (OPM)^10^ uses a single-objective lens (referred to as the primary objective) for both illumination and detection, without additional reflecting elements at the sample space. The fluorescence is first relayed to a secondary objective, then relayed to a tertiary objective and finally focused onto a camera. This configuration trades optical complexity for sample mounting simplicity, that is additional optical elements (including two remote objective lenses) are added on the detection path so that there is only one objective at the proximity of the sample. This ‘single-objective’ light-sheet microscope configuration is compatible with diverse specimens including the intact rodent brain^11^ and multi-well plated samples^12,13^. As a drawback, OPM traditionally was unable to use the full numerical aperture (NA) of the primary objective for fluorescence detection, lowering its resolution and sensitivity. This has been addressed more recently via refractive index-mismatched remote focusing^13–15^, which unlocks the potential of OPM for high-resolution imaging by giving it access to the full NA for detection. However, full NA detection imaging has only been demonstrated with high magnification systems (×100 and ×60), for a limited field of view up to 200 μm^15^. This field of view cannot accommodate large living samples such as developing embryos and fundamentally limits imaging throughput. The standard solution to this problem is to tile the acquisition over multiple fields of view, sacrificing imaging speed.

Another limitation of light-sheet microscopy when applied to large living specimens such as zebrafish embryos is the presence of structures in the sample that absorb, refract and scatter both the illumination and detection light. An effective solution is multi-view imaging, where samples are illuminated and detected from different orientations and images with better coverage and overall quality can be computationally reconstructed^1,16–19^. However, multi-view imaging often requires either complex multi-objective configurations that complicate sample mounting, or sample rotation that decreases imaging speed.

**Fig. 1:**
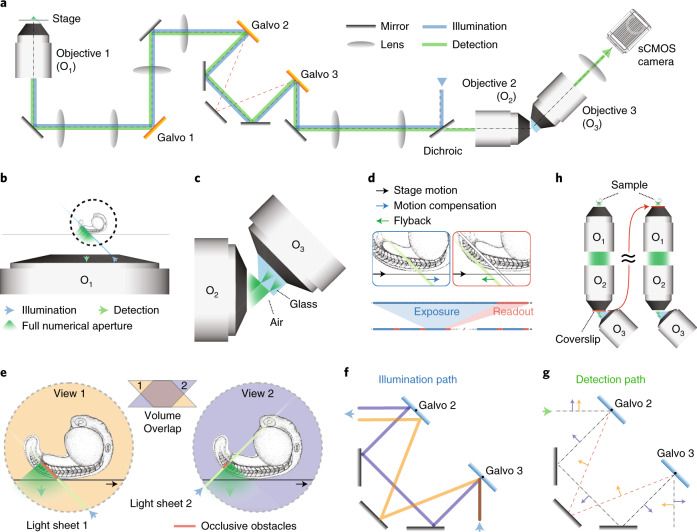
Design of a high-resolution, large field of view and multi-view single-objective light-sheet microscope. **a**, Simplified scheme of the optical setup. **b**, In this setup, the light-sheet excitation and emission pass through a single objective. The fluorescence is collected by O_1_ and relayed downstream with full NA detection, ensuring high-resolution imaging. **c**, The full NA detection is achieved by oblique remote focusing using a bespoke objective with a monolithic glass tip and zero working distance. The glass tip compresses the collection half-angle allowing a tilt range from 0 to 55°. **d**, During imaging, the stage moves the sample along the scanning axis. To avoid motion blur, the galvo mirror moves the light sheet alongside the stage movement during the camera exposure for each image. The galvo mirror moves back during the readout time and restarts this compensatory movement during the next exposure. Illumination and detection planes remain centered along the entire optical train to give optimal light collection, minimal aberrations and thus pristine image quality. **e**, Our instrument is capable of dual light-sheet excitation. This improves illumination coverage and image contrast, as for most points in the sample, one of the two light-sheet orientations will have a shorter penetration depth through the sample giving a more contrasted and complete image. The dual-view imaging is achieved through an imaging flipping module consisting of two galvo mirrors and three normal mirrors along the optical path (**f** and **g**). **f**, The illumination light goes along the path highlighted in orange or blue, resulting in opposing incident angle at the sample space. **g**, Similarly, the fluorescence light goes through either of the two paths, resulting in the flipping of the image with respect to that of the other path (blue and orange arrows before and after propagation through the unit), ensuring that the intermediate image is always formed on the front surface of O_3_. **h**, The microscope is converted from upright (dipping, left side) to inverted (immersion, right side) by repositioning the coverslip from the focal space of O_2_ to that of O_1_, without sacrificing the optical performance.

**Extended Data Fig. 1:**
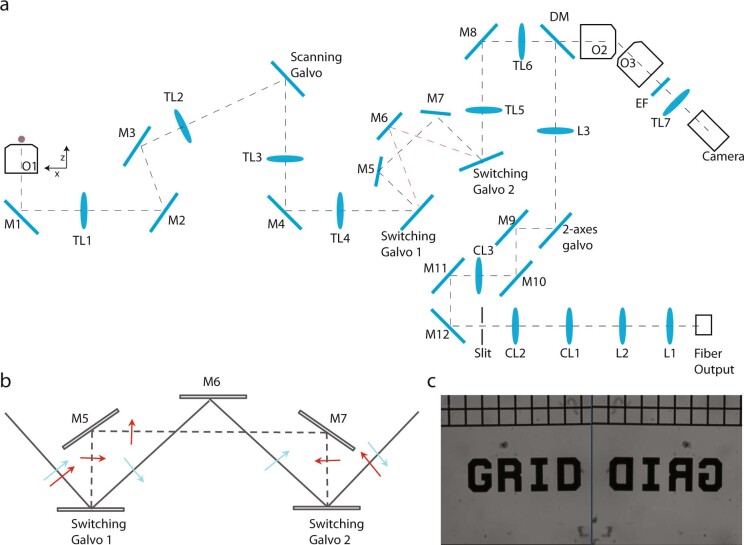
Optical setup of the microscope. (**a**) Detailed layout of the setup. Objectives lenses: O_1_ - Olympus XLUMPLFLN 20XW, O_2_ - Olympus UPLXAPO20X, O_3_ - Calico AMS-AGY v2.0. Tube lenses: TL1, TL2 and TL3 - Olympus SWTLU-C 180 mm, TL4, TL5 and TL6 - 135 mm custom designed tube lens (using off-the-shelf pieces from Thorlabs, see [ref]), TL7- Thorlabs TTL200-A or TTL165-A. Scanning galvo and switching galvos: Cambridge 6SD12205 20 mm galvo mirrors. Cylindrical lenses: CL1-CL3. Achromatic double lenses: L1-L2. Dichroic mirror: DM - Chroma ZT405/488/561/640rpcv2-UF3. Mirrors: M1-M12, protected-silver coated mirror. 2-axes galvo: Cambridge 6SD12056 10 mm galvo mirrors. EF: emission filters, Chroma ET525/50 m or ET605/75 m. (**b**) Dual view switching module. The two switching galvo mirrors can switch the light path so that light is reflected either by M5 and M7 or by M6 only. The red arrow is reflected four times along the path and maintains upright, while the green arrow is reflected only three times and becomes inverted. (**c**) Dual view images of a calibration grid (Thorlabs R1L1S1P). The two images are clearly flipped with respect to each other. The images were taken under bright field illumination and with O_3_, TL7 and the camera on a straight line with O_2_.

To address both of these problems, we present DaXi: a new single-objective design that is capable of imaging large samples, with a large imaging volume, uncompromised image resolution and speed, and multi-view imaging. *Da* and *Xi* are both words from Chinese Mandarin, where *Da* refers to the large imaging volume and *Xi* for the high-resolution capability. DaXi achieves this goal by means of several innovations (Fig. 1, Extended Data Fig. 1, Supplementary Figs. 1–7 and Notes 1 and 2). First, we use a new custom tertiary objective that maintains the image resolution and field of view of a low-magnification primary objective (×20). Second, we introduce a new fast three-dimensional (3D) scanning modality—light-sheet stabilized scanning (LS3)—that further extends the effective imaging volume (that is, scanning range) without compromising imaging speed or quality. Third, we show how to achieve multi-view imaging and enhance volumetric coverage and image quality with dual illumination. Fourth, we further improve sample mounting ergonomics by converting our microscope from upright to inverted using remote focusing. Last, we demonstrate these new capabilities by imaging *Drosophila* egg chamber development, zebrafish tail development, whole-brain activity in zebrafish larvae and multiple zebrafish embryos in a high-throughput format.

## Results

### Larger field of view and high resolution

Our first goal when designing our microscope was to achieve high resolution over a large field of view. In OPM, a single-objective lens (referred to as the primary objective) is used for both illumination (oblique) and fluorescence collection (Fig. 1b). The fluorescence is relayed to a secondary objective with aberration-free remote focusing^20^. A tertiary objective is arranged at the same angle as the illumination light-sheet tilting angle with respect to the secondary objective, to focus the fluorescent signal from the obliquely illuminated imaging plane onto a camera (Fig. 1c). The secondary and tertiary objectives are both mounted remotely, leaving only the primary objective at the proximity of the sample. This configuration makes the optical system effectively a ‘single’ objective light-sheet microscope, thus facilitating sample mounting and handling.

To maintain the spatial resolution and the field of view of the upstream optical system, the tertiary objective needs to have high NA (at least 1.0), adequate field of view and reasonable mechanical dimensions to avoid collision with the secondary objective. Previously, both water-immersion^13^ and glass-tipped^14,15^ objectives have been demonstrated to fulfill such requirements for high magnification systems (×100 and ×60) with a field of view up to 220 μm. To extend the field of view for low-magnification systems (×20 in this work), we used a custom tertiary objective (AMS-AGY v.2.0) with an NA of 1.0 and a field of view up to 750 μm. This custom tertiary objective is the second instance of a family of objectives that are specifically designed for single-objective light-sheet microscopy (Fig. 1c, Supplementary Note 3 and Supplementary Figs. 5–7). It features an air–glass imaging boundary and a working distance of zero for maximum mechanical clearance and hemispherical collection in air, thus allowing our optical system to achieve uncompromised high resolution with a large field of view.

### Light-sheet stabilized stage scanning

**Extended Data Fig. 2:**
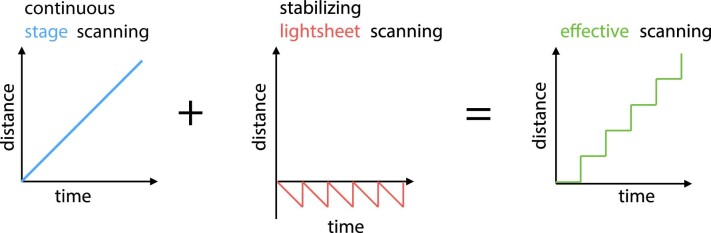
Light sheet stabilized stage scanning (LS^3^). During the acquisition of a 3D image stack, the stage moves continuously, and the galvanometer scanner performs a counteracting motion of the light-sheet and detection planes to cancel out any relative motion between sample and imaging plane (which in our design is always coplanar with the light-sheet illumination), resulting in an effective step-wise scan of the sample.

In light-sheet microscopy, a large imaging volume not only requires a large field of view but also a long scanning range as the sample is imaged one plane at a time. Our next challenge was the choice of scan method. Volumetric scanning in a single-objective light-sheet microscope is done either by moving the imaging plane (both the illumination and detection) or the sample relative to the other. Moving the imaging plane is faster because it can be implemented with a fast actuator such as a galvo scanner. However, the volumetric scanning range is limited by the field of view of the primary objective. Alternatively, one can perform tiled imaging by combining galvo scanning with stage movement between tiles. However, this lowers temporal resolution because of the hardware settling time between tiles (Supplementary Fig. 8). Another disadvantage is that tiled acquisition requires additional postprocessing to obtain a stitched image, and tiling artifacts often cannot be entirely suppressed. Moreover, when using galvo scanning per tile, the imaging quality worsens away from the objective’s optical axis because of the optical limits of remote focusing, objectives and galvanometric scanning. In contrast, moving the sample relatively to the imaging plane solely by moving the microscope’s stage does not suffer from these shortcomings and offers a scanning range of more than 10 mm. However, stage scanning by stepwise motions has limited temporal resolution, and continuous stage scanning is fast but can potentially introduce motion blur. Due to OPM’s scan geometry, stage scanning introduces both axial and lateral blur. This is problematic as the lateral resolution is typically much better than the axial resolution and hence more sensitive to blur. Multiple methods have been used to mitigate this issue. For example, strobing the illumination light can reduce motion blur at the cost of increased peak illumination intensity. One can also move the stage slowly so that the motion is limited within one camera frame, again at the cost of temporal resolution. To solve the various challenges raised by stage and galvo scanning while retaining their key advantages, we developed LS^3^, which combines the high-speed of a galvo scanner and the large scan range of a microscope stage (Fig. 1d and Extended Data Fig. 2). The stage moves continuously while a compensatory motion of the imaging plane induced by a galvo scanner cancels out any relative motion between the sample and the imaging plane during the exposure of one camera frame. The imaging plane is then brought back quickly to the starting position during the camera readout and before a new frame is acquired. All advantages of stage scanning are retained (Supplementary Table 1), while simultaneously benefiting from the advantages of high-speed galvo-based scanning. Considering the typical exposures and travel speeds needed in practice, the only true limiting factors of the scanning speed are camera speed and fluorophore brightness.

### Dual light-sheet illumination for multi-view imaging

With the large imaging volume achieved, we are ready to image large samples. The next challenge we faced was that large samples have occluding, refracting and scattering structures that could reduce image quality. To improve the optical coverage and to have consistent image quality, we optically alternate the light-sheet illumination and the viewing direction (orthogonal to the light sheet) around ±45 degrees with respect to the optical axis and imaged the sample sequentially to give a pair of complementary orthogonal views, as shown in Fig. 1e–g. This is achieved by using two galvo mirrors to quickly alternate between two optical paths. One path has two additional mirrors, while the other path adds only one (Fig. 1f,g, orange and purple paths), hence effectively flipping the light with respect to the optical axis. This image flipping module affects both illumination and detection plane simultaneously so that the imaging plane always falls on the glass surface of the tertiary objective regardless of whether the illumination light goes through the sample from left or right. Consequently, both views share the same downstream and upstream optical path, largely simplifying the optical setup and avoiding additional cost. The orthogonal image planes will benefit from the contrasting trajectories into the sample, in many cases avoiding obstacles and therefore returning complementary information that can be fused in postprocessing.

### Remote coverslip enables inverted microscopy

**Extended Data Fig. 3:**
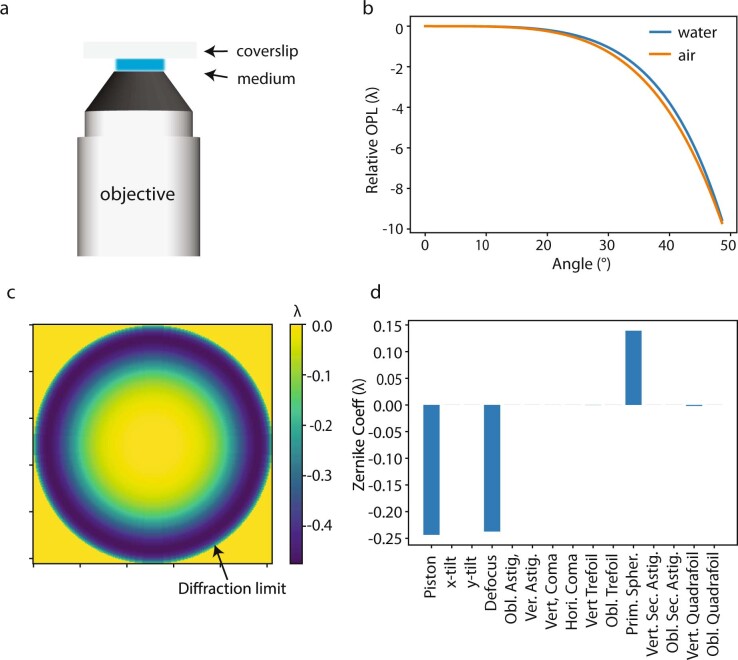
Analysis of the optical path length when placing a glass coverslip into the imaging medium of an objective. (**a**) illustrates the optical system to analyze. Depending on the type of objective, the medium could be air, water, immersion oil, etc. (**b**) plots the optical path length of an emitter at the coverslip surface as a function of the angle between the emission ray and the optical axis, in the case of air or water medium. (**c**) shows that 2D map of the optical path difference of using air and water medium. This 2D phase map is then fitted with the first 50 Zernike terms. (**d**) shows the coefficients of the first 20 Zernike terms. The major non-zero Zernike terms are piston, defocus and primary spherical aberration. Since the piston and defocus terms are trivial, this suggests that the significant aberration is primary spherical and is most likely introduced when the coverslip is moved from the secondary objective (air) to the primary objective (water) in a remote focus system (see Fig. 1). Considering that in practice the primary spherical aberration can be compensated by moving any of the relay tube lenses, one should be able to maintain the optical performance of the microscope when switching the coverslip’s location. Simulation is performed for objectives of NA 1.0 water and NA 0.75 air. The Zernike fitting is done using pyOTF, a simulation software package for modeling optical transfer functions (OTF)/point spread functions (PSF) of optical microscopes written in python. Link: https://github.com/david-hoffman/pyOTF.

**Extended Data Fig. 4:**
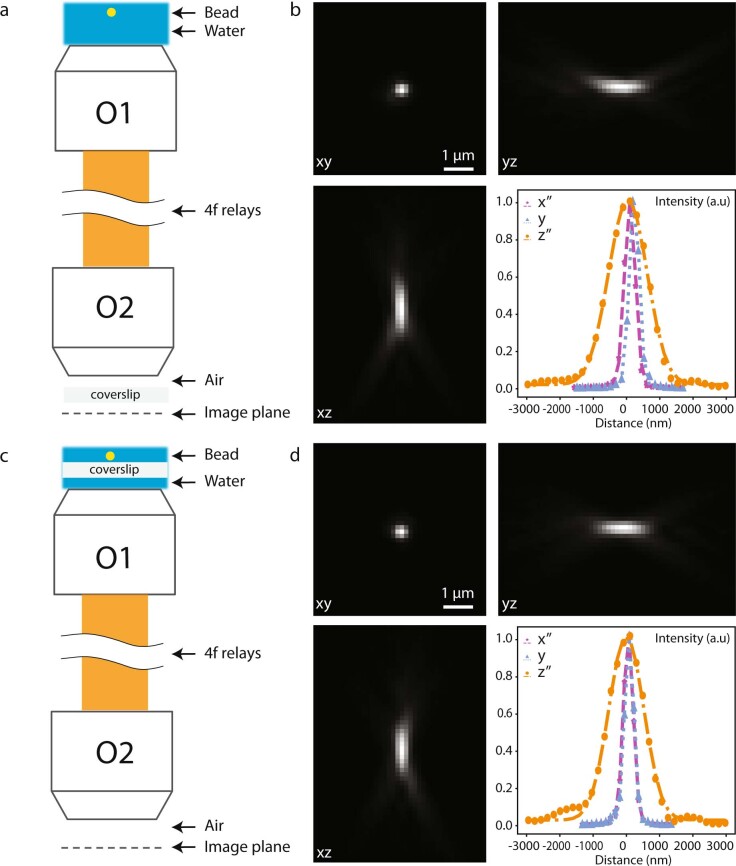
Converting the microscope from upright to inverted by repositioning the coverslip in a remote focusing system. (**a**) shows the remote focusing system composed of two objectives whose pupil planes are conjugated by a 4 f relay system. Yellow dot: fluorescent bead. O_1_: 20x, 1.0NA, water dipping. O_2_: 20x, 0.8NA, air. According to their factory design, O_1_ should be used directly facing the sample without any coverslip in between. O_2_ requires a coverslip between the objective front lens and the image plane, as shown in (a). This configuration is well suited for an upright microscope where the primary objective is used in a dipping configuration. (**b**) shows a representative PSF of the setup in (a) measured with 100 nm green fluorescence beads. The average FWHMs are respectively 400.5 + - 16.9 nm (x), 349.9 + - 20.4 nm (y) and 1356.8 + - 97.4 nm (z), n = 7 beads. Interestingly, according to our analysis (see Extended Data Fig. 3), one can reposition the coverslip at the focal space of O_2_ to that of O_1_ as shown in (c) and can still achieve similar optical performance, turning the system to an inverted configuration. (d) shows a representative PSF of such a system (c). The average FWHMs are respectively 382.2 + - 8.9 nm (x), 376.4 + - 22.0 nm (y), 1353.6 + - 48.6 nm (z), n = 6 beads. The numbers are indeed similar to that of the top configuration (a).

Our last consideration was to ensure ease and flexibility of sample mounting, which is often restricted because even single-objective systems are either configured for immersion or dipping states. For example, in our instrument, the primary objective (Olympus XLUMPLFLN 20XW) does not require a coverslip, whereas the secondary objective (Olympus UPLXAPO20X) does (left side of Fig. 1h). This configuration is suitable for upright imaging where the primary objective can be dipped into the imaging medium directly without needing to image through a glass coverslip. However, we discovered that the coverslip needed by the secondary objective can be moved to support the sample (right side of Fig. 1h), effectively turning this system into an inverted configuration. Moreover, this was achieved without affecting the image quality according to simulations and experimental results (Extended Data Figs. 3–4). Our lens configuration considerably simplifies microscope design and increases its versatility: inverted imaging enables many imaging modalities, including multi-well plates and microfluidic devices, as well as on-stage sample manipulation.

### Instrument characterization

**Fig. 2:**
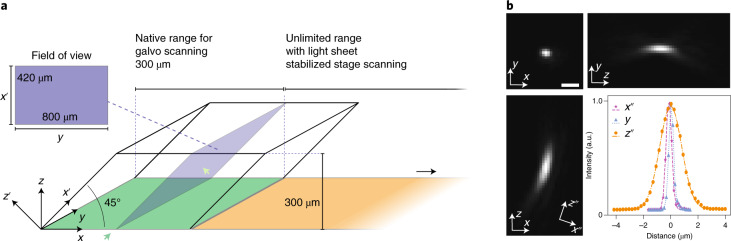
Characterization of the microscope. **a**, Imaging volume geometry. The coverslip is parallel to the *xy* plane. The optical axis of the microscope is along the *z* axis (depth). The sample is illuminated by an oblique light sheet in the *x*’*y* plane, where *x*’ is the light-sheet propagation direction. The field of view in the *x*’*y* plane is 800 μm (*y*) by 420 μm (*x*’), corresponding to 800 μm (*y*) by 300 μm (depth, *z*) in the *yz* plane. Volumetric data were acquired by scanning the sample, along the *x* axis, with respect to the illumination plane. By using light-sheet stabilized stage scanning, the scanning range (up to 75 mm, compared to 300 μm with galvo scanning) is only limited by the stage. **b**, Representative PSF obtained by imaging 100-nm green fluorescence beads. Projections along *xy*, *xz* and *zy* are shown. The PSF is slightly tilted and its long axis (*z*”) is about 20° with respect to the *z* axis. Taking this into consideration, the line profiles of the PSF were plotted and fitted along the three principal axes, that is *x*”, *y* and *z*”. The FWHM are, respectively, 479.9 ± 28.0, 379.2 ± 20.9 and 1,864.9 ± 174.3 nm (mean ± s.d., *n* = 156 fluorescence beads). Scale bar, 1 μm.

**Extended Data Fig. 5:**
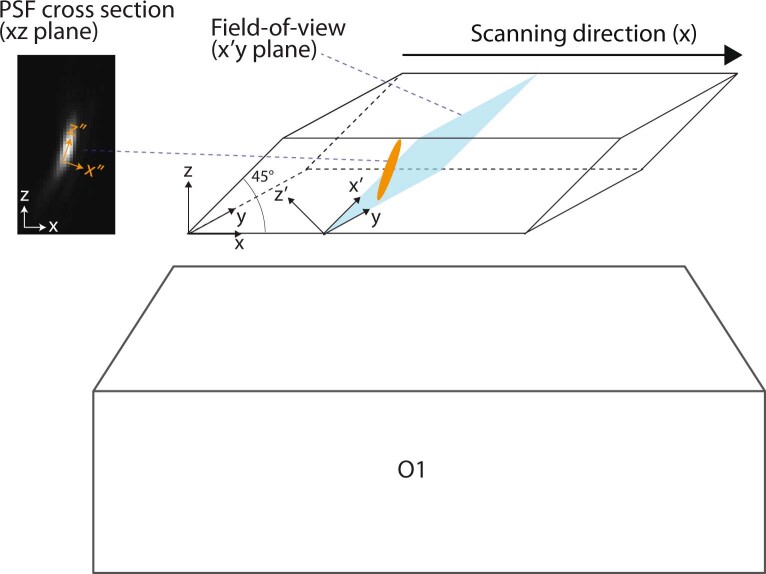
Coordinate system of the microscope. The objective front lens is parallel to the xy plane. The optical axis of the microscope is along the z axis (depth). The sample is illuminated by an oblique light sheet in the x’y plane, where x’ is the light sheet propagation direction. Volumetric data were acquired by scanning the sample, along the x-axis, with respect to the illumination plane. Because the PSF is slightly tilted, that is, and its long axis (z”) is about 20° with respect to the z-axis. The line profiles of the PSF were plotted and fitted along the three principal axes (x”, y, and z”) to measure the FWHMs.

**Extended Data Fig. 6:**
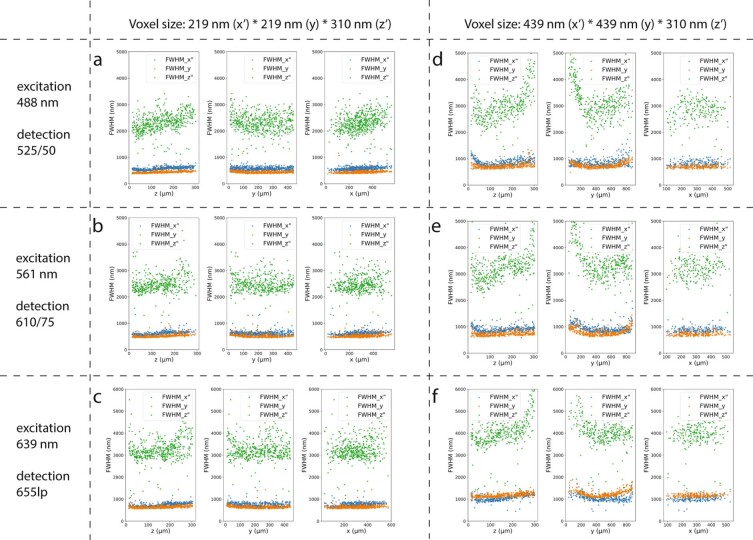
PSF measurements across the imaging volume. In order to have a good sampling of the PSF for accurate estimation of the resolution, we set the effective magnification to 29.6 and the pixel size to 220 nm. With the chip size of our camera being 2048 * 2048, we can achieve a field of view 451 μm (y) * 451 μm (x’). This corresponds to 451 μm (y, width) * 319 μm (z, depth) in the sample coordinates. The scanning range doesn’t affect the imaging quality and can go as far as the stage’s moving range. We then acquire images of 100 nm fluorescence beads at three channels and plot the FWHMs along the three axes of the PSF. Note that the PSF is slightly tilted with respect to the xyz coordinates. Therefore, we perform the FWHMs measurements along the principal axes to give a better estimation of the lateral resolution (x”, y) and especially the axial resolution (z”). This avoids underestimation of the true axial resolution. The left column (**a-c**) shows the plots of the FWHMs within the imaging volume. The performance is consistent, up to ~ 300 μm depth for all three channels. For large field of view imaging, we can use a different tube lens (TL7, Extended Data Fig. 1) so that the effective magnification is 14.8 and the pixel size is 440 nm. We then achieve a field of view of 901 μm (y) * 451 μm (x’) using half of the camera chip (2048 * 1024). This corresponds to 901 μm (y, width) * 319 μm (z, depth) in the sample coordinates. The right column (**d-f**) shows the FWHMs across the imaging volume under this configuration. Overall, the PSFs are consistence but they do get widens towards the edge of the field of view. See also Table 1 for the statistics of the FWHMs. Source data

We first measured the optical performance of our instrument. The imaging volume is width × 800 × 300 μm (*x*, *y* and *z*, respectively, Fig. 2a and Extended Data Fig. 5) where the width can span multiple milimeters. The raw camera frames are in the *x*’*y* plane, and the field of view is 800 μm (*y*) by 420 μm (*x*’), corresponding to 800 μm (*y*) by 300 μm (depth, *z*) in the *yz* plane. Volumetric acquisition is achieved by scanning the stage (along the *x* axis) with the light sheet stabilized relative to the sample to avoid motion blur. Scanning using a galvo has a limited range of about 300 μm, beyond which the illumination starts to be cropped and most importantly the imaging performance starts to degrade. The custom tertiary objective guarantees high spatial resolution by full NA detection, thus the nominal NA of the microscope is close to that of the primary objective O_1_ (that is, 1.0). In practice, we note that the light is compressed toward one edge of the pupil of the tertiary objective O_3_ along the *x*_0_ axis due to the air–glass interface between O_2_ and O_3_ (Supplementary Figs. 9 and 10). As a result, the effective pupil function is no longer symmetric with respective to the optical axis along the *x*’ axis. We measured the point-spread function (PSF) using 100-nm green fluorescence beads in both views. The full-width at half-maximum (FWHM) values of the PSF along the three principal axes are 479.9 ± 28.0, 379.2 ± 20.9 and 1,864.9 ± 174.3 nm, respectively (Fig. 2b). Some aberrations remain in the optical system that mostly originate from the primary objective. This affects the PSF, especially under oblique remote focusing. Future designs that incorporate a deformable mirror could potentially correct system aberrations and further improve the PSF. Further analysis of the PSF reveals that the FWHM are consistent across the imaging volume, for three different color channels, for two magnifications and under stage scanning with LS^3^ (Extended Data Fig. 6 and Tables 2 and 3). The increased detection NA of our microscope gives better spatial resolution but also a much higher detection signal, which is important when imaging at high temporal resolution and at low photodamage regimes (Supplementary Fig. 11).

**Fig. 3:**
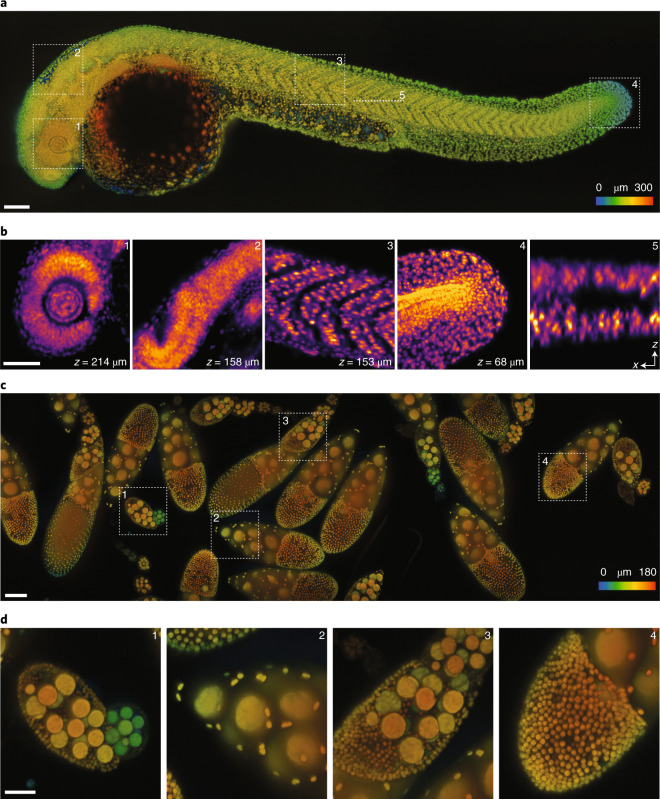
Large volume imaging of *Danio rerio* larval development and *Drosophila melanogaster* egg chambers. **a**, Images of a zebrafish larvae (roughly 30 hpf, nuclei labeled with tg(h2afva:h2afva-mCherry) imaged using the microscope. Imaging volume (*x*,*y*,*z*) is 3,000 × 800 × 300 μm acquired every 50 s (two views). The depth is color-coded, where blue and red indicate respectively close to and far from the coverglass. Scale bar, 100 μm. **b**, Four *xy* slices from different regions (1–4, dashed squares in **a**) at various depths and one *xz* slice (5, dashed line in **a**) are highlighted. Scale bar, 50 μm. **c**, Images of *Drosophila* fly egg chambers. Nuclei of germline cells (large) and somatic cells (small) were labeled by expressing UAS-NLS-GFP under the control of Usp10-Gal4 (BDSC-76169). Imaging volume is 3,000 × 800 × 180 μm acquired every 30 s (single views) for 3 h. The depth is color-coded as above. Scale bar, 100 μm. **d**, Four regions (dashed squares in **c**) are highlighted. Scale bar, 50 μm.

**Extended Data Fig. 7:**
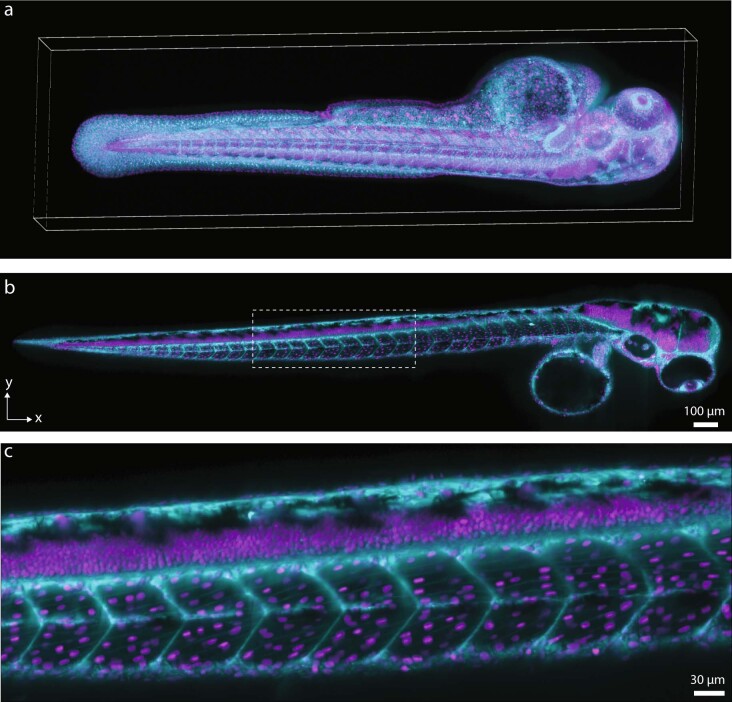
Dual color imaging of a zebrafish larvae. The larvae is imaged at 2 dpf. The nuclei (magenta) are labelled with tg(h2afva:h2afva-mCherry). The membranes (cyan) are stained using Vybrant DiO cell-labeling solution (Thermal fisher V22889). Dio injections for retrograde live label was applied at 24 hpf, followed by an O/N incubation at 29 C incubator before imaging. (**a**) 3D volume rendering of the data. (**b**) Representative xy slice of the 3D data. (c) Magnified view of the region highlighted in the white dashed rectangle in (b).

**Extended Data Fig. 8:**
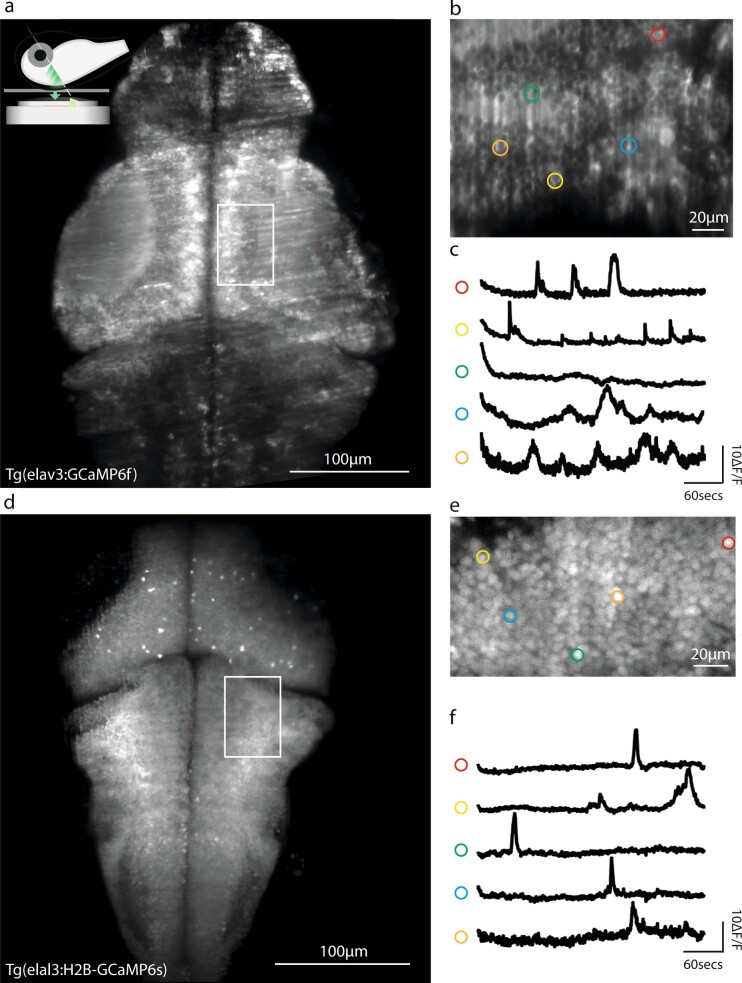
Whole-brain, neuron-level 3D imaging in larval zebrafish in vivo. High-resolution images are recorded in steps of 8 μm with an exposure time of 8 ms. A volume of 500 μm * 300 μm * 200 μm, containing the entire brain, is recorded once every 0.3 s. The recording of two different zebrafish lines is shown in (a)-(c) and (d)-(f). (**a**) and (**d**) show the max intensity projection of the 3D volumes. (**b**) and (**e**) show regions of interest from a single slice of the 3D stack, indicated by the write rectangles in (a) and (d). (**c)** and (**f**) give representative fluorescence signal of five different neurons over time.

**Extended Data Fig. 9:**
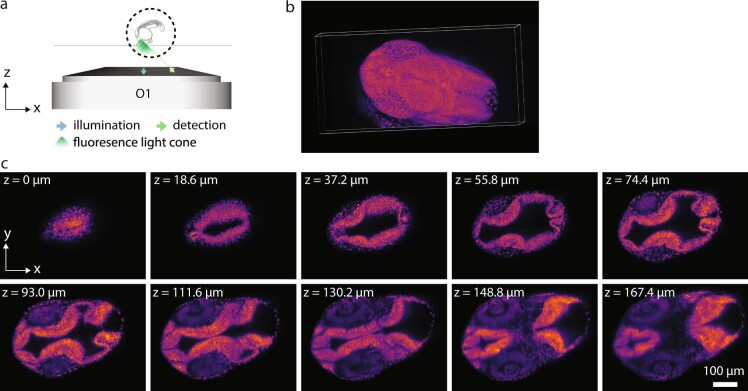
Imaging the whole brain of the zebrafish embryo. (**a**) show the orientation of the embryo mounted on the sample stage, with the head facing the primary objective (O_1_). (**b**) 3D rendering of the zebrafish brain images. (**c**) XY slices of the brain images at different depths up to 167.4 mm.

Our instrument achieves a combination of large imaging volume and spatial resolution, demonstrated by imaging of whole zebrafish larvae (Fig. 3a,b, Supplementary Video 1 and Extended Data Fig. 7) and *Drosophila* fly egg chambers (Fig. 3c,d and Supplementary Video 2). These images are obtained from single 3D stack acquisitions and do not require stitching, corresponding to an imaging volume of 3,000 × 800 × 300 μm. The dimension along the scanning direction (*x* axis) is only limited by the scanning range of the stage (in our case 75 mm). LS^3^ is key to acquiring such large volumes at high quality. However, when imaging smaller samples (limited to about 300 μm), galvo scanning is faster because its settling time after one volumetric scan is just a few milliseconds, compared to hundreds of milliseconds for the stage. Our instrument is capable of both scanning modes, providing flexibility dependent on the desired volume dimensions. The imaging speed largely depends on the scanning range, but also on signal level, scanning step size and so on. For an imaging volume of 2,000 × 800 × 300 μm, we typically achieve an imaging speed of about 10 to 30 s per volume. By tuning imaging parameters according to experimental requirements, much higher speeds are achievable, similar to other single-objective light-sheet implementations^13,21^. For instance, we imaged zebrafish larvae whole-brain activity using genetically encoded calcium indicators at a 3.3 Hz volumetric imaging rate (Extended Data Fig. 8). Adapting the mounting strategy to the sample is necessary to achieve optimal imaging quality. For example, the embryo in Fig. 3a is placed sideways with its body axis parallel to the coverglass to image the whole embryo. In the case of functional brain imaging, one should instead mount the embryo with the head facing the coverglass (Extended Data Fig. 9) so that both hemispheres of the brain are equally accessible.

### Imaging zebrafish tail development

**Fig. 4:**
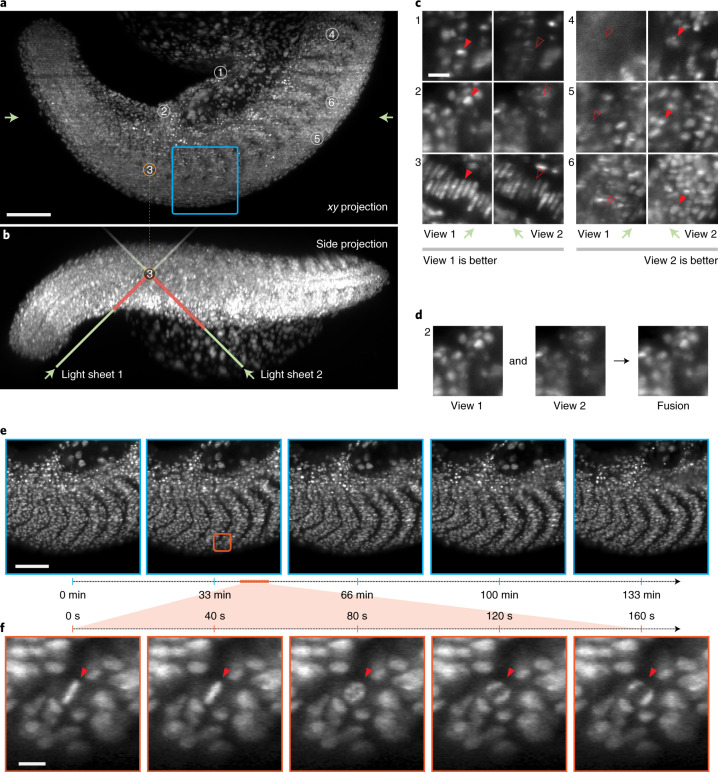
High-speed multi-view imaging of zebrafish tail development. **a**, Axial maximum projection showing the whole zebrafish larva tail at 24 hpf, nuclei labeled with tg(h2afva:h2afva-mCherry). Imaging volume is 1,064 × 532 × 287 μm consisting of 4,000 × 2,000 × 360 voxels per view for a total of 5.7 billion voxels acquired every 40 s. Scale bar, 100 μm. **b**, Side projection illustrating how the two light-sheets enter the sample at 45° to reach a given point in the sample. Depending on the sample geometry and placement, one of the two light-sheets will have a shorter path to reach that point and hence be less susceptible to absorption, refraction or scattering. Consequently, the corresponding view’s image will be more complete and better contrasted. **c**, Example regions (single *xy* plane slices) that demonstrate the complementarity of the two views. In some regions (left) the first view has better image quality, whereas in other regions (right) the second view is better. Scale bar, 3 μm. **d**, After registration, the two views can be fused together to obtain one high-quality image. **e**, Time-lapse max-projection frames over a 2.2 h period centered on the dorsomedial tail, during which time the boundary between neighboring somites are accentuated. Scale bar, 80 μm. **f**, Spatio-temporal zoom centered around a cell division, single *xy* plane slice. Despite the large field of view, both views are acquired every 40 s making it possible to follow the intermediate steps during mitosis—an important capability for achieving, for example, accurate lineage tracking. Scale bar, 10 μm.

Together, the high resolution, large imaging volume and multi-view features of DaXi enabled us to follow the zebrafish tail development at high spatio-temporal resolution over many hours. For example, we imaged the tail extension of a 24 hpf (hours postfertilization) embryo for 8 h. To minimize mechanical stress around the animals and allow their normal development, embryos are embedded in exceptionally soft (0.1%) agarose gel in a glass-bottom petri dish. Sample holding stability during imaging mainly comes from the gravity of the sample itself as well as agarose viscosity. Figure 4a,b shows that the whole tail can be imaged from the posterior part of the tail (the tail bud) to the anterior trunk, spanning more than a millimeter of tissue along the anterior–posterior axis. The two views obtained by dual orthogonal illumination maximized coverage and image quality. Indeed, as shown in Fig. 4c these views are complementary in terms of contrast and coverage. Depending on the region inspected, one view or the other will be better. In general, we find a good agreement between our observations and our assumption that given a point in the sample, the longer the light-sheet penetration depth, the poorer the image quality for the corresponding view. For each time point we obtain a single fused image (Fig. 4d) consisting of 4,000 × 2,000 × 360 voxels. Each fused volume was acquired in 40 s, providing sufficient temporal resolution to closely follow cell division (Fig. 4e,f). This spatio-temporal resolution and imaging volume is important because many applications in developmental biology require the ability to track cells and follow lineages of the whole animal—something that is very difficult or in fact nearly impossible if cells divide faster than the acquisition speed.

We also recorded somitogenesis during zebrafish development (from 10 to 18 hpf), as well as tail extension (from 24 to 32 hpf) for 8 h (Supplementary Figs. 12 and 13 and Supplementary Videos 3 and 4). The imaging volume was up to 2,200 × 800 × 300 μm, with each volume captured at 30-s intervals. With our microscope, we can follow the distinct stages of zebrafish tail development at high spatio-temporal resolution for 8 hours. Longer imaging sessions are conceivable and only limited by the effectiveness of the sample immobilization protocol. To further demonstrate the spatio-temporal resolution and large imaging volume of DaXi for applications in developmental biology we performed nuclear segmentation and tracking of a zebrafish embryo between 12 and 18 hpf over its optically accessible regions (Supplementary Video 5).

### Imaging nine zebrafish embryos

**Fig. 5:**
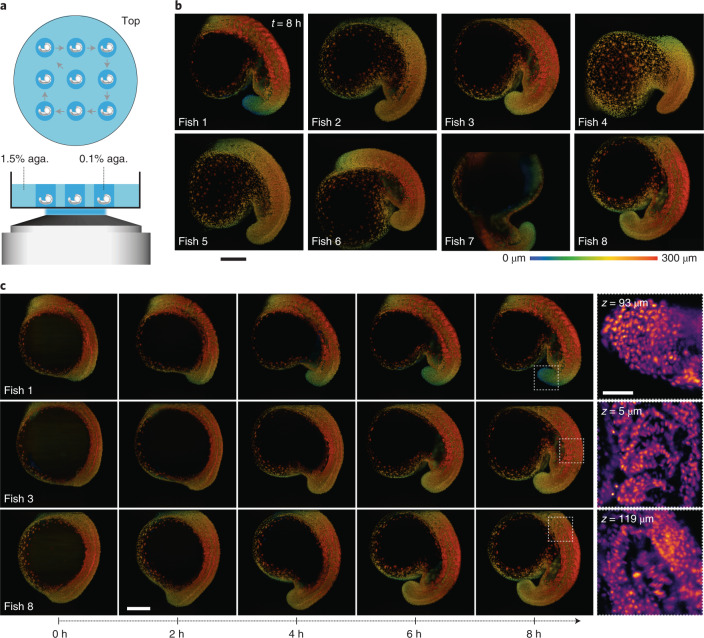
Imaging nine zebrafish embryos at a time. **a**, Top and side views of nine zebrafish embryos mounted in 0.1% agarose gel. **b**, The embryos (only eight are shown) were imaged sequentially (at 4.5 min per round) for up to 8 h. Only the final frames at *t* = 8 h imaging are shown (see also Supplementary Video 6 for the time lapse of all nine embryos). All the embryos developed normally. The images are maximum intensity projection, color-coded for depth, of the 3D volume. Scale bar, 200 μm. **c**, Five time points from three different fish are shown, illustrating the imaging reproducibility across multiple samples. Scale bars, 50 μm (top right) and 200 μm (bottom left).

Last, the inverted configuration of the microscope is compatible with multi-well imaging that facilitates sequential imaging of multiple samples. Previous attempts at simultaneously imaging multiple samples in a large imaging volume and multi-view light-sheet microscope required the mounting of up to five embryos in FEP (fluorinated ethylene propylene) tube sections assembled using FEP connectors^22^. While successful, this approach is inherently nonscalable and unpractical as it forces a delicate sample mounting protocol on the user. In contrast, single-objective light-sheet microscopes let users reuse standard sample mounting protocols already developed for standard microscope systems (for example, widefield and confocal). Here, as a proof of concept, we mounted nine embryos in 0.1% agarose gel in a glass-bottom petri dish (Fig. 5a) and imaged them sequentially (at roughly 4.5 min per round) for a total of 8 h (Fig. 5b,c and Supplementary Video 6). The samples were mounted in an orientation that allowed us to focus on the embryos’ posterior development. Overall, the simultaneous imaging of the nine samples was very reliable and provided extremely good resolution, allowing us to follow developmental processes such as somitogenesis in each embryo at single cell resolution (Supplementary Video 7). This mounting strategy is easily scalable, allowing for simultaneous imaging of multiple samples and high-throughput screening.

## Discussion

We have designed, built and characterized DaXi, a single-objective light-sheet microscope that can achieve high spatio-temporal resolution over large imaging volumes and long scanning range without introducing motion blur or sacrificing imaging speed. Moreover, our microscope allows for improved illumination coverage through dual illumination. We achieved a resolution of roughly 450 nm laterally and 2 mm axially, and an imaging volume of 3,000 × 800 × 300 mm. The first dimension is only constrained by the stage travel range, which is 75 mm with our stage. Previous OPM systems with almost full NA detection^13,15^ reported a field of view of 220 × 60 mm. Here we demonstrated a field of view about 18 times larger, at the cost of slightly reduced resolution. Compared to SCAPE2.0^21^ and SoPi^23^, which use the same primary objective and report an effective detection NA of 0.35, our microscope achieves almost full NA detection of approximatively 0.97 through refractive index-mismatched remote focusing, covering a similar field of view. To compare the optical throughput of different state-of-art OPM methods, we calculate the modified etendue (Supplementary Table 4)—DaXi achieves at least 2.6 times more throughput than previous systems.

Our light-sheet scanning approach LS^3^ extends the scanning range of the system further by more than tenfold, while maintaining high temporal resolution without inducing motion blur. Suppression of scattering and shadowing artifacts is achieved by illuminating and detecting views from two orthogonal directions, following a similar rationale to other multi-view light-sheet microscopes^16,17^ but implemented in a single-objective geometry. We note that Sparks et al. independently reported another method for dual-view imaging in OPM by translating a pair of tilted mirrors in a folded remote focusing setup^24^. In this setup, light is lost due to the beam-splitter needed for recombining both paths, and the mechanical scanning of the mirrors lowers the switching speed. Our approach has the advantages of high switching speed, uncompromised light efficiency and modularity as it can be added to other microscopes. We demonstrate the capabilities of our microscope by imaging large samples, including zebrafish larvae and *Drosophila* fly egg chambers, without the need for tiled acquisition. The versatile mounting strategies, low phototoxicity of light-sheet illumination and high spatio-temporal resolution of the microscope design allow us to image zebrafish embryo development for 8 h at 30-s time intervals. In combination, these features will allow investigators to precisely follow cell fate and lineages with improved accuracy, pushing the limit of imaging to enable the capture of complex cellular choreography during early embryonic development.

Crucially, DaXi achieves this performance without forcing unconventional sample mounting procedures on the user—especially when compared with standard multi-view light-sheet microscopes^17,19^. We introduce the remote coverslip method to enable a more versatile inverted configuration that is compatible with many imaging modalities. Biologists can now mount samples including multi-well plates in a convenient manner, prepare multiple specimens to image only the best, image multiple specimens in parallel and flow drugs and other chemicals with microfluidics, while maintaining a sterile environment. The free space above the sample can potentially be leveraged to combine imaging with other sensing or manipulation schemes, such as patch clamping or mechanical perturbation. We demonstrate parallel imaging of nine samples in this paper as a proof of principle. The number of samples that can be imaged or screened can be largely increased using a multi-well mounting protocol^12,13^. Augmenting our system with a microfluidics sample mounting system would enable large-scale screens of mutants or drug-treated animals.

Many of the concepts introduced in this paper are also applicable to other imaging modalities. The remote coverslip method can easily convert objective lenses between immersion and dipping using remote focusing, expanding their use^25^. LS^3^ can also be used in other light-sheet systems to speed up imaging and to increase the scanning range by adding a fast actuator such as a galvo to actuate the light sheet. Dual illumination further improves the overall image quality by providing alternate illumination and viewing angles. Potentially, image rotation by other angles below or beyond 180° could add even more illumination and detection diversity. The dual-view image flipper is not limited to OPM with full NA detection, but is also applicable to other variants of OPM^11,26–28^.

The spatial resolution of the microscope can be further improved through adaptive optics^29,30^ to correct both the system and sample-induced aberration. Currently, the two views are fused through content-aware blending. It is also possible to further improve the resolution through multi-view deconvolution^24,31,32^. The axial resolution could also be further enhanced using an axially scanned and thus thinner light sheet^8,33^. However, this reduces the duty cycle of the excitation, which could restrict its suitability for long-term live-sample imaging.

Given its versatility, we expect the microscope described here to be broadly adopted by researchers and applied to a wide range of multi-scale and high-throughput imaging applications. This includes imaging organoids, which are particularly suited to multi-well imaging and cleared tissue^33,34^, and for which the high spatio-temporal resolution and large imaging volume would prove to be critical. Future directions for development, including smart microscopy and data processing with machine learning, will further improve the optical performance of the system, the versatility and ease of use of the microscope, and the data processing efficiency and quality.

## Methods

### Microscope description

Extended Data Fig. 1 shows the detailed optical setup. A primary objective (O_1_, Olympus XLUMPLFLN 20XW NA1.0, water) is used to both generate an oblique light sheet in the sample and to collect the fluorescence. A series of tube lenses (TL1 to TL6) conjugate the pupils of O_1_ and O_2_ so that an intermediate image of the sample at O_1_ is formed at the secondary objective O_2_ (Olympus UPLXAPO20X). The intermediate image has a uniform magnification of 1:33, equal to the refractive index ratio of the medium of O_1_ versus that of O_2_, making the optical train aberration-free over a reasonable volume^20,35^. A custom tertiary objective O_3_ (AMS-AGY v.2.0, Supplementary Figs. 5–7 and Supplementary Note 3) is oriented by 45° with respect to O_2_. The fluorescence is filtered by either individual bandpass filters (Chroma ET525/50, ET605/70) or a quad-band filter (Chroma ZET405/488/561/640) and then detected by a scientific complementary metal-oxide semiconductor (sCMOS) camera (Hamamatsu ORCAFlash 4.0). The pixel size of the cameras at the sample space is 146 nm (TL7—Thorlabs AC300A) for PSF calibration and 265 nm (TL7—Thorlabs TTL165-A) or 440 nm (TL7—Thorlabs TTL100-A) for imaging so as to capture the desired field of view. Objective O_3_ is mounted on a piezo stage (PI Fast PIFOC Z-Drive PD72Z1SAQ) so that its focus can be finely adjusted.

Two switching galvo mirrors are used to create two views of the object in the sample space. By adjusting the angles of the two galvo mirrors, the light is reflected either by just one or by two reflective mirrors. The operating principle is similar to that of a Dove prism. Moreover, the switching module also changes the incident angle of the light sheet between +45° and −45° since the excitation light also passes through this module. Illumination and detection scanning. A galvo mirror (Cambridge Tech, 20 mm galvo, 6SD12205) is conjugated to the pupil planes of both O_1_ and O_2_. Rotating the galvometer actuated mirror scans the oblique light sheet across the sample (along the *x* axis), with the incident angle kept at 45°. The galvo mirror also descans the intermediate image at the focal space of O_2_ so that the intermediate image is always projected at the focal plane of O_3_. Using the galvometer for 3D scanning allows for faster imaging compared to stage scanning, but at the cost of a limited scan range of approximately 300 µm.

A motorized stage (ASI MS-2000) is used to position the sample and to perform scanning for volumetric imaging. During acquisition of each frame, stage scanning is combined with galvo descanning to stabilize the imaging plane (coplanar light sheet and detection planes) relative to the sample. In the absence of relative motion between the sample and the imaging plane, no motion blur occurs. Stabilized light-sheet stage scanning allows for much longer ranges compared to galvometer-based scanning. It is only limited by the range of travel of the stage, in our case 75 mm. Illumination and detection planes remain fixed and optimally placed at the center of O_1_ and O_2_ native axes thus guaranteeing optimal light collection, minimal aberrations and thus optimal image quality.

For long-term imaging, a water dispenser was built to automatically supply immersion water between the primary objective and the sample (Supplementary Fig. 3). The water dispenser consists of a micropump (part of a Leica Water Immersion Micro Dispenser), a microcapillary tip (Eppendorf Microloader) and a 3D-printed objective cap. An environmental chamber (Supplementary Fig. 4) was built around the sample to keep it at roughly 28 °C.

Supplementary Notes 1 and 2 provide a more detailed description and alignment procedure, Supplementary Fig. 1 contains photos of the actual setup, Supplementary Fig. 2 has an image of the computer-aided design model of the microscope, Supplementary Video 8 shows an animation of the setup and Supplementary Table 5 contains a the list of components used to construct the microscope.

### Optical setup characterization

The microscope’s PSF was measured using 100-nm green fluorescence beads. The beads are first embedded in agarose gel (0.5%) and then deposited on a glass coverslip (no. 1.5). The resulting images are then deskewed to the objective-aligned frame of reference (*xyz* coordinates). The positions of the beads are then detected using by finding the local maxima and a cropped image of each bead is used for further analysis. To account for the tilted PSF with respect to the *z* axis, the cropped images are rotated in the *xz* plane so that the long axis of the PSF (*z*” axis) is along the *z* axis. The PSFs are then fitted with a one-dimensional Gaussian function along all three axes (*x*”, *y* and *z*”). The values of the FWHM are then averaged from all fluorescence beads in the imaging volume.

### Light-sheet incident angle adjustment and stripe reduction

A two-axis galvo mirrors (Cambridge 10 mm 6SD12056) are conjugated with the sample plane so that rotating the two mirrors results in a rotation of the excitation beam at the sample plane. In particular, the incident angle of the light sheet at the focal space of O_1_ can be adjusted by one of the mirrors to 45° with respect to the optical axis. The effective excitation NA is estimated to be 0.08 at this incident angle. The effective excitation NA can be potentially increased, either through reducing the incident angle of the light sheet or using objectives with higher NA. Increasing excitation NA would allow generating a thinner light sheet to further improve the axial resolution but at the expense of field of view^36–38^. To reduce the stripe due to sample absorption and obstruction of the illumination light, the light sheet is swept continuously from −6° to 6° (Supplementary Fig. 14) in the illumination plane during the acquisition of each frame, using the other mirror of the two-axis galvo.

### Microscope control software

The data acquisition and display is done by the open-source, freely available software Micro-Manager 2.0 Gamma^39^. A custom-developed micromanager script sets up the acquisition order and stores the hyperstack data in TIFF files. A NI DAQ system programmed using a Python module controls the timing of all devices during acquisition. The NI DAQ system consists of one compact chassis (cDAQ-9178), two analog control modules (NI 9263 4-Channel AO) and one digital control module (NI 9401 8-channel DIO). The Python module uses the NI-DAQmx Python API to program the DAQ system. It synchronizes the devices, including camera, motorized stage, galvo mirrors and lasers during data acquisition. Alternatively, one can also use Pycromanager^40^ to perform data acquisition in the Python environment instead of the Java environment of Micro-Manager. The water dispenser is controlled by another Python module to supply water to the primary objective during long-term recordings.

### Image processing library: dataset exploration and processing

All data processing is done using our open-source Python package dataset exploration and processing (dexp). This library performs a number of image processing tasks specific to light-sheet imaging including equalization, denoising, dehazing, registration, fusion, stabilization, deskewing and deconvolution. It leverages napari^41^ for multi-dimensional visualization and 3D rendering, CuPy^42^ for graphical processing unit- (GPU-) accelerated computing, Dask^43^ for scalable array computing and zarr^44^ as multi-dimensional data storage format.

### Image processing pipeline

**Extended Data Fig. 10:**
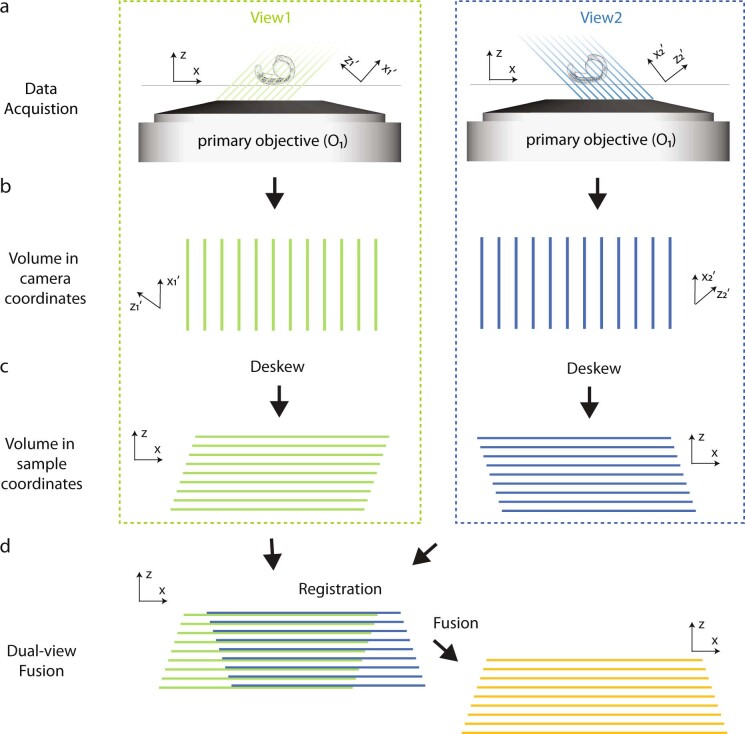
Data processing pipeline. The 3D stacks from each view are initially in the x_1_’yz_1_’ and x_2_’yz_2_’ coordinates respectively. The stacks are then resampling to the sample (that is, xyz) coordinates. This process involves resampling of the voxels from the x_1_’yz_1_’ (or x_2_’yz_2_’) coordinates to the xyz coordinates (see Extended Data Fig. 5 and see Supplementary Fig. 15). The two stacks are then registered and fused to a single stack (see Supplementary Fig. 16).

The multi-dimensional data are saved by Micro-Manager to local storage of the control PC in TIFF format. The TIFF files are then read out and converted to zarr format where typically a five- to tenfold data compression is achieved through lossless compression. The zarr datasets are then transferred to a local server equipped with 200 TB storage and 4 NVIDIA Tesla V100 SXM2 32 GB for further processing. The data processing pipeline is shown in Extended Data Fig. 10. Each 3D stack from one of the views is deskewed to coverslip-based coordinates, that is the *xyz* coordinates (Supplementary Fig. 15). The images are then dehazed to remove large-scale background light caused by scattered illumination and out-of-focus light. The image from the second view is then registered to the first view using an iterative multi-scale warping approach (Supplementary Fig. 16). In each iteration, the images are divided into chunks along all three axes by a factor of 2*i* where *i* is the current iteration number; corresponding chunks from both views are registered separately with a translation model to produce a translation vector; a vector field is then calculated based on all the translation vectors and the image of the second view is warped according to the vector field. This procedure repeats until the maximum number of iterations or the minimal size of the chunk is reached. In this work, the maximum iteration number was set to four and the minimal chunk size 32 × 32 × 32. This procedure results in a spline-interpolated vector field that is applied to the second view. After registration, images from the two views are fused by picking regions from one or the other image based on the local image quality^45^. The magnitude of the Sobel gradient was used as the metric to generate a blend map, based on which the two images are blended. Other fusion methods such as frequency domain fusion (discrete cosine transform, fast Fourier transform) are available in dexp. An illumination intensity correction is applied to the fused data to account for the Gaussian profile of the light sheet along the *y* axis. Each raw data per time point is around 8 GB (two views, roughly 1,000 × 1,024 × 2,048 × 2 voxels). An 8-hour continuous imaging session produces roughly 8 TB of raw data (roughly 1,000 time points). It takes roughly 1 min to process the data per time point per GPU and 4 h to process the whole dataset with four GPUs running in parallel. After all time points are processed, they are temporally stabilized to compensate minor drifts over time.

### Temporal stabilization

Temporal stabilization is performed by computing for each time point, the relative displacement vector to its 2 × *n* neighbors, where *n* is typically chosen to be 7. The displacement vector between two 3D images is simply computed by phase correlation. Once all these relative shifts are obtained, we formulate and solve the linear system: *R* = *M***A** in which **A** is the vector of ‘absolute’ positions of the sample, *M* is the band matrix that encodes the relationship between absolute and relative shifts and *R* is the vector of observed relative shifts. The goal is to recover the absolute positions **A** from the relative measurements *R*. A simple approach is to simply invert this linear system in a least-square sense. However, in practice the measurements that constitute *R* can be affected by noise, and so we use L_1_ regularized inversion to obtain the best results. The code can be found at https://github.com/royerlab/dexp/blob/master/dexp/processing/registration/sequence.py.

### Video rendering

Color max-projection rendering is done using code implemented as part of our dexp library (https://github.com/royerlab/dexp). Some of the more complex volume rendering videos involving rotations are done with napari-animation^41^ (https://github.com/royerlab/dexp): an easy to use napari plugin (https://napari.org/) capable of complex keyed animation.

### Segmentation and tracking

The nuclei segmentation and tracking are computed jointly using the approach presented by Türetken et al.^46^. First, the 3D cell boundaries are predicted using a neural network^47^. The predicted cell boundaries are then fed to a hierarchical watershed algorithm from Higra^48^ (github.com/higra/Higra), to generate an initial set of segments by accounting for partial occlusions and overlaps. Last, the tracking algorithm then picks the optimal segments that maximize the total integer programming cost^46^. We define the individual integer programming cost to connect the segments between adjacent time points as the intersection over union of their masks. The flow fields of the nine fish tails were produced by smoothing the tracklets with Savitzky–Golay filtering and coloring them with their track identifiers, which is correlated with the time point of their appearance. The tracks of the whole embryo were colored with the orientation of the flow field^49^.

### Sample preparation

Zebrafish husbandry and experiments were conducted according to protocols approved by the UCSF Institutional Animal Care Use Committee. In the experiments, we used tg(h2afva:h2afva-mCherry (a gift from J. Huisken, Morgridge Institute for Research) and for the functional imaging of neuronal activity, we used tg(elevl3:GCaM6f) and tg(elavl3:H2B-GCaMP6s)^50^. The sample mounting geometry is shown in Supplementary Figs. 17 and 18. First, zebrafish were dechorionated with a pair of sharp forceps underneath a binocular dissecting microscope and incubated for at least 5 min in a solution of fish water and tricaine (0:016%). Embryos were gently pipetted into a 0:1% solution of low gelling point agarose (Sigma, A0701) cooled at 37 °C. The embryos, together with approximately 1 ml of 0:1% agarose, were placed in a glass-bottomed petri dish (Stellar Scientific catalog no. 801001). Using a capillary needle, the embryo was gently positioned at the center of the dish and in the desired orientation: laterally in this case. When the agarose was solidified, the dish was flooded with fish water and 0:016% tricaine. All time-lapse experiments were done with a gentle flow of embryo medium water with 0.016% tricaine at 28 °C, using peristaltic pumps, allowing normal development of the embryo and meanwhile preventing embryo movement. However, occasionally it was possible to observe sudden embryo movements during recordings that resulted in image artifacts: for example, one in Supplementary Video 3 (at roughly 4 min of video time and roughly 63 min of experiment time) and multiple occasions in Supplementary Video 4.

For the calcium imaging experiment fish were embedded in 2% agarose (Sigma, A0701) and anesthetized in an external solution with tricaine (0.2 mg ml^−1^) at 5 dpf.

For *Drosophila* fly imaging, the egg chambers were dissected and cultured as described previously^51^. *D. melanogaster* ovaries were removed from females 3 d after eclosion for observation. Fly stocks Usp10-Gal4 (BDSC-76169) and UAS-GFP.nls (BDSC-4776) were used. The egg chambers were then transferred to a glass-bottomed petri dish for imaging. The mounting was similar to that used for the zebrafish imaging, except that the egg chambers were immersed in imaging media (Schneider’s media supplied with 200 μg ml^−1^ insulin, 15% (vol/vol) FBS, 0.6× penicillin–streptomycin, pH 6.95–7.0.) rather than agarose solution.

### Imaging conditions

Imaging conditions for all experiments can be found at Supplementary Table 6.

### Statistics and reproducibility

Each experiment was repeated three times for Fig. 3a, 17 times for Fig. 3c,d, eight times for Fig. 4a–f and Supplementary Fig. 12, three times for Fig. 5b,c, five times for Extended Data Fig. 7, three times for Extended Data Fig. 8, three times for Extended Data Fig. 9 and seven times for Supplementary Fig. 13.

### Reporting Summary

Further information on research design is available in the Nature Research Reporting Summary linked to this article.

## Online content

Any methods, additional references, Nature Research reporting summaries, source data, extended data, supplementary information, acknowledgements, peer review information; details of author contributions and competing interests; and statements of data and code availability are available at 10.1038/s41592-022-01417-2.

## Supplementary information

Supplementary Information Supplementary Figs. 1–25, Notes 1–3 and Tables 1–6.

Reporting Summary

Supplementary Video 1 Rotating rendering of a *D. rerio* (zebrafish) embryo at 30 hpf imaged on DaXi. Illumination wavelength 488 nm. 3,000 × 800 × 300 µm × 1 view. Voxel size 0.440 × 0.440 × 1.806 µm. Single stack acquisition lasted 52 s.

Supplementary Video 2 DaXi imaging of egg *D. melanogaster* (fly) egg chambers 3 d after eclosion. Transgenic line UAS-GFP.nls (BDSC-4776). Illumination wavelength 488 nm. 3,000 × 800 × 300 µm × 1 view. Voxel size 0.440 × 0.440 × 1.806 µm. Acquisition lasted for 2.5 h with an acquisition interval of 27 s for a total 342 time points.

Supplementary Video 3 DaXi imaging of a *D. rerio* (zebrafish) embryo starting at roughly 10 hpf and ending at 18 hpf. Transgenic line h2afva.mCherry. Illumination wavelength 561 nm. 2,200 × 800 × 300 µm × 2 views. Voxel sizes 0.440 × 0.440 × 1.806 µm. Acquisition lasted 9 h with a 30-s acquisition interval for a total of 1,079 time points.

Supplementary Video 4 DaXi imaging of a *D. rerio* (zebrafish) embryo starting at roughly 24 hpf and ending at 32 hpf. Transgenic line h2afva.mCherry. Illumination wavelength 561 nm. 3,000 × 800 × 300 µm × 2 views. Voxel sizes 0.440 × 0.440 × 1.806 µm. Acquisition lasted 8 h with 73-s acquisition interval for a total of 390 time points.

Supplementary Video 5 DaXi imaging, nuclei segmentation and cell tracking of a *D. rerio* (zebrafish) embryo starting at roughly 12 hpf and ending at roughly 18 hpf. Transgenic line h2afva.mCherry. Illumination wavelength 561 nm, 2,200 × 800 × 300 µm × 2 views. Voxel sizes 0.440 × 0.440 × 1.806 µm. Acquisition lasted 6.6 h with 30-s acquisition interval for a total of 791 time points.

Supplementary Video 6 High-throughput DaXi imaging of nine *D. rerio* (Zebrafish) embryos starting at roughly 10 hpf and ending at roughly 16 hpf. Transgenic line h2afva.mCherry. Illumination wavelength 561 nm. 1,000 × 800 × 300 µm × 2 views. Voxel sizes 0.440 × 0.440 × 1.806 µm. Acquisition lasted 4.6 h with 167.5 s per fish per stack for a total of 100 time points.

Supplementary Video 7 High-throughput DaXi imaging, nuclei segmentation and cell tracking of nine *D. rerio* (Zebrafish) embryos starting at roughly 10 hpf and ending at roughly 16 hpf. Transgenic line h2afva.mCherry. Illumination wavelength 561 nm. 1,000 × 800 × 300 µm × 2 views. Voxel sizes 0.440 × 0.440 × 1.806 µm. Acquisition lasted 4.6 h with 167.5 s per fish per stack for a total of 100 time points.

Supplementary Video 8 Video abstract illustrating the main concepts behind DaXi: (1) the sample mounting advantages of single-objective light-sheet microscopy for high-throughput imaging, (2) an explanation of stabilized light-sheet scanning (LS3), (3) multi-view imaging for improved coverage, (4) uncompromised resolution with the AMS-AGY v.2.0 ‘Snouty’ custom objective, (5) high-fidelity 3D rendering of the whole microscope and optics setup and (6) the innovations (animation and rendering by L.W. Whitehead).

## Data Availability

A sample data of early zebrafish embryonic development, which is useful for testing the dexp processing software, can be found at github.com/royerlab/dexp. Source data are provided with this paper.

## Source data

Source Data Extended Data Fig. 6 Source data in Excel format.

## Extended data

are available for this paper at 10.1038/s41592-022-01417-2.

## Code availability

All code can be found at github.com/royerlab/daxi. These include the Python code for optical simulation, device control and the Micro-Manager acquisition script. Our image processing tool dexp can be found at github.com/royerlab/dexp.
