# Supplementary material for: DaXi—high-resolution, large imaging volume and multi-view single-objective light-sheet microscopy
Source: Nat Methods. 2022 Mar 21;19(4):461–9. doi: 10.1038/s41592-022-01417-2 (PMC9007742; doi:10.1038/s41592-022-01417-2)
Supplement: Supplementary file 1 — Supplementary Figs. 1–25, Notes 1–3 and Tables 1–6. [file 41592_2022_1417_MOESM1_ESM.pdf]

nature portfolio

https://doi.org/10.1038/s41592-022-01417-2

Supplementary information

DaXi—high-resolution, large imaging volume and multi-view single-objective light-sheet microscopy

In the format provided by the authors and unedited

Supplementary Information
DaXi – High-Resolution, Large Imaging Volume, and Multi-View Single-Objective Light-Sheet Microscopy

Bin Yang[1]*, Merlin Lange[1], Alfred Millett-Sikking[2], Xiang Zhao[1], Jordão Bragantini[1], Shruthi VijayKumar[1], Mason Kamb[1], Rafael Gómez-Sjöberg[1], Ahmet Can Solak[1], Wanpeng Wang[3], Hirofumi Kobayashi[1], Matthew N. McCarroll[4], Lachlan W. Whitehead[5,6], Reto P. Fiolka[7,8], Thomas B. Kornberg[3], Andrew G. York[2], Loic A. Royer[1]*

1 Chan Zuckerberg Biohub, San Francisco, USA
2 Calico Life Sciences LLC, South San Francisco, USA
3 Cardiovascular Research Institute, University of California, San Francisco, CA 94143
4 Department of Pharmaceutical Chemistry, University of California, San Francisco, CA, 94143, USA.
5 The Walter and Eliza Hall Institute of Medical Research, Parkville, VIC, Australia
6 Department of Medical Biology, The University of Melbourne, Parkville, VIC, Australia
7 Department of Cell Biology, University of Texas Southwestern Medical Center, Dallas, United States
8 Lyda Hill Department of Bioinformatics, University of Texas Southwestern Medical Center, Dallas, United States

* Correspondence: loic.royer@czbiohub.org, bin.yang@czbiohub.org

Supplementary Note 1. Optical setup

Extended Data Fig. 1a shows a detailed scheme of the optical setup of the microscope. A primary objective (O1, Olympus XLUMPLFLN 20XW NA1.0, water) was used to both generate an oblique light sheet in the sample and to collect the fluorescence. A series of tube lenses (TL1 - TL6) conjugate the pupil pupils of O1 and O2 so that an intermediate image of the sample at the focal space of O1 was formed at the focal space of the secondary O2 (Olympus UPLXAPO20X). The intermediate image has a uniform magnification of 1.33, equalling to the refractive index ratio of that of O1 and O2, so that it is aberration-free. A tertiary objective O3 (Calico AMS 2.0) is oriented by 45° with respect to O2. The fluorescence was filtered by either individual bandpass filters (Chroma ET525/50, ET605/70) or a quad-band filter (Chroma ZET405/488/561/640) and then detected by a scientific complementary metal-oxide semiconductor (sCMOS) camera (Hamamatsu ORCAFlash 4.0). The pixel size of the cameras at the sample space was 147 nm (TL7 - Thorlabs AC300-A) for PSF calibration, 220 nm (TL7 - Thorlabs TTL200-A) and 440 nm (TL7 - Thorlabs TTL100-A) for imaging in order to capture the desired field of view. O3 was mounted on a piezo stage (PI Fast PIFOC Z-Drive PD72Z1SAQ) so that its focus could be finely tuned.

Illumination. The illumination light came out of a fibre output which input is from a custom laser combiner of two lasers (Vortran Stradus 488 nm and 561 nm). The light is firstly collimated by a telescope composed of two achromatic lenses (L1- L2) and then expanded along the horizontal direction by two cylindrical lenses (CL1-CL2). It is further focused on to the 2-axes galvo mirrors by CL3 and then reflected by a dichroic mirror (Chroma ZT405/488/561/640rpc) to be combined with the detection path. The 2-axes galvo mirrors (Cambridge 10 mm 6SD12056) are conjugated with the sample plane so that rotating the two mirrors resulted in a rotation of the excitation beam at the sample plane. In particular, the incident angle of the light sheet at the focal space of O1 can be adjusted by one of the mirrors to 45_ with respect to the optical axis. The effective excitation NA is estimated to be about 0.08.

Optical reflector. The two switching galvo mirrors shown in Extended Data Fig. 1b can create two views at the remote space of the object at the focal space of O1. By adjusting the angles of the two galvo mirrors, the light is reflected either by M5 and M7 or by M6 only. The operating principle is like that of a Dove prism. The red arrow is reflected one more time compared to the green arrow. As a result, it is flipped along one direction compared to the green one. The intermediate image of the sample is therefore flipped along the horizontal plane. Extended Data Fig. 1c shows two images of a calibration grid taken with the two views, and it clearly shows that the image in the right view is flipped along the horizontal axis compared to the image shown on the left. Moreover, the switching module also changes the incident angle of the light sheet between +45° and -45° since the excitation light also passes through this module. Instead of using galvo mirrors, one can also consider using a mirror mounted on a

motorised stage to send the light to different paths. We chose galvos mirrors for these purposes since they are often at least one order of magnitude faster than motorized stage. It is also possible to have a mirror fold system mounted on a rotating mount. By rotating this module, one would be able to create more than two views of the sample, with in principle unlimited possible views.

Imaging plane scanning. A Galvo mirror (Cambridge Tech, 20mm galvo, 6SD12205) was conjugated to the pupil planes of both O1 and O2. Rotating the Galvo mirror scanned the oblique light sheet across the sample (along the x axis), with the incident angle kept at 45°. The Galvo mirror also descanned the intermediate image at the focal space of O2 so that the intermediate image was always projected at the focal plane of O3. Using the galvo for image scan allows faster imaging speed compared to stage scanning. The scanning range is limited to about 300 µm, both due to cropping of the excitation beam by the relay tube lenses and decreased optical performance when the illuminated plane is away from the optical axis of O1.

Supplementary Note 2. Optical system construction and alignment procedure.

Test samples

1. A multi-frequency grid distortion target (Thorlabs, R1L1S1P) to measure the magnification of the system.

2. A #1.5 glass coverslip (170 µm thickness) uniformly coated with the fluorescent dye (e.g. fluorescein) to characterize the light sheet.

3. 100 nm fluorescent beads embedded in 2% agarose gel to characterize the resolution of the system.

General remarks and guidelines for alignment

1. There are a few planes are conjugated with 4f-systems in the optical setup. Firstly, the pupil planes of O1, O2, the scanning galvo mirror and the slit are conjugated. The conjugation between the pupil planes of O1 and O2 ensures aberration-free imaging; the conjugation between the scanning galvo mirror and the pupil plane of O1 ensures constant incident angle of the oblique light sheet and proper image descanning; the conjugation between the pupil plane of O1 and the slit allows adjusting the light sheet thickness. Secondly, the 2-axes galvo mirrors and the focal plane of O1 are conjugated so that the incident angles of the light sheet can be adjusted.

2. O1, O2 and O3 are all mounted on translation stages (Thorlabs XR25P) so that their position can be precisely adjusted.

3. Start by placing and aligning all the mirrors at their corresponding locations (either through estimation or follow a CAD design).

4. Then place the lenses one by one, using a reflective mirror at the sample plane to adjust the lateral positions, check the light collimation (with a shear interferometer (Thorlabs SI050) or other means) to adjust the axial positions.

5. Lastly fine tune the system to have diffraction-limited imaging, mostly by assuring proper conjugation between critical planes.

Detailed alignment procedure

<u>Set up all optics</u>

1. Setup the mount for the fibber collimator at desired height.

2. Place mirrors (including the dichroic mirror) after the collimator one by one, ensures that the height is constant, and the beam hits the centre of the mirrors. Note that the height is different before and after the two-axes galvo mirrors. Ignore M6 for now, but make sure that the switching galvos are set to the correct value and their value can be adjusted to send light to approximately the centre between M5 and M7.

3. Place a few irises (Thorlabs ID25 and ID50) along the path to aid alignment of lateral positions of the lenses.

4. Make sure that the light passes through the mount of O1 cantered.

5. Place a reflective mirror at the place of O1, slightly adjust M1 so that the reflected beam passes all the iris cantered. Go back to step 4 if necessary.

6. Place L1 and L2 to have a collimated beam.

7. Place TL1, adjust its lateral position so that the reflected beam passes all the iris, and its axial location is approximately at the desired location.

8. Place TL2, adjust its lateral position so that the reflected beam passes all the iris, adjust its axial position so that the beam is collimated after TL1.

9. Repeat step 8 for TL3 to TL6.

10. Start with having O2 and O3 along a straight line. Make sure the reflected light passes through the centre of the mounts of O2 and O3, then place O2 and O3 in the setup.

11. Place TL7 in the setup, make sure that the light passes through its centre.

12. Place the camera in the setup, depending on the specificity of TL7, make sure the distance from TL7 to the camera sensor is correct.

13. Place O1 in the setup.

Adjust the position of O1 and O2 to have them both conjugated to the galvo mirror.

14. Send a sinus wave signal to the scanning galvo, adjust axially O1 so that the light coming out of it is scanning laterally without changing angles. This is the desired position for O1.

15. Put the grid sample on the sample stage. If necessary, add a spacer beneath it so that O1 is approximately at its desired position.

16. Use a flashlight to illuminate the grid and have its image on the camera.

17. Move O3 200 µm away/closer to O2, then measure the magnification of the system with the grid. The goal is to have the same magnification regardless of the relative position of O3 to

O2. Move O2 and O3 together axially until at one point the magnification is uniform. The pupil plane of O2 is then conjugate to that of O1.

18. Translate the stages to move O2 and O3 together axially until at one point the magnification is uniform.

19. Place a sample with beads imbedded in 0.5% agarose on the sample stage. Place the emission filter, switch on the laser and observe the fluorescence image of the beads. The defocused image of the beads should look circular, otherwise slightly adjust O2 laterally to have a circular image of the beads. Adjust O3 slight to bring back the field of view to the centre of the camera. One can move O3 axially to inspect the images of the beads along different z. The image quality should be constant across at least 500 µm, otherwise the alignment is subject to further trouble shooting and improvement.

Dual view component alignment.

20. Adjust the voltages sent to the switching mirrors so that the light is reflect to the centre of the M6 and also passes through the irises from M6 to O1 thought the centre.

21. Place the grid back to the sample stage and measure the magnification with O3 at 200 µm away and closer to O2. Translate the stage to move M6 so that the magnification is uniform when translating O3 axially.

22. Repeat step 20.

Oblique light sheet setup and alignment.

23. Place O3 and the components downstream at 45° to O2. Place a sample with beads imbedded in 0.5% agarose on the sample stage. Adjust O3 so that the fluorescence image of the beads is capture by the camera in the centre of the field of view.

24. Set up CL1 and CL2 so that the laser light is expanded along the direction horizontal to the optical table.

25. Set up CL3 so that the beam is focused on the 2-axes galvo.

26. Place the slit at approximately the focal plane of CL3.

27. Place L3 so that the light becomes collimated again along the vertical direction.

28. Place a sample with beads imbedded in 0.5% agarose on the sample stage. Adjust the sample of the y-axes galvo to adjust the incident angle of the light sheet to 45° within the xy plane. Adjust O3 slightly to refocus if necessary. When the light sheet is at the correct angle, all beads in the image will appear in focus.

Supplementary Note 3. AMS-AGY v2.0 Objective – Full technical details and context.

Light-sheet fluorescence microscopy (LSFM) or selective plane illumination microscopy (SPIM) is a powerful technique for biological imaging. Fast, gentle, and with good sectioning, the idea has seen much attention with innovations like the DiSPIM[1] and Lattice[2], and commercial instruments like the Zeiss Lightsheet 7 and the Leica DLS. However, traditional light-sheet designs require two or more orthogonal lenses for illumination and collection, resulting in an awkward interface between the biology and the optics, and a major drawback for many users and applications.

The Oblique Plane Microscope (OPM)[3] invention of 2008 restored the traditional coverslip boundary by passing light-sheet excitation and emission through a single primary objective, and then using a tilted remote refocus (RR) in the downstream optics to image the equally tilted plane of illumination (the object plane). OPM showed that light-sheet microscopy could be done with a standard microscope and sample interface, but seemingly exchanged this convenience for heavy losses in resolution and optical efficiency.

The problem with OPM was that the tilted portion of the remote refocus would lose a significant fraction of the emission light, simply because the numerical aperture of the final objective was too low. An ideal objective for this location would collect all the emission light, even with the additional tilt imposed by the OPM architecture, i.e. a full hemisphere of collection (a seemingly impossible requirement when objective half angles are typically limited to 70 deg).

However, in 2018 the Epi-illumination SPIM microscope (eSPIM)[4] showed that the major optical losses in OPM style systems could in fact be avoided. By using a water objective and coverslip assembly as the final objective, the numerical aperture could now equal the refractive index of air (1.0) i.e. the 'immersion medium' of the opposing objective in the remote refocus. This is the crucial insight: to make the elusive 'hemisphere' collection objective you simply need a numerical aperture that is greater than (or equal to) the index of the medium in which it operates. So for example, an NA 1.0 water immersion objective has a modest 49 deg collection cone in water (a reasonable lens to manufacture), but in air this transforms to 90 deg. So by imaging at the coverslip boundary, a water lens can indeed collect a solid angle of 2pi from an air medium. There are however some additional considerations that complicate the eSPIM approach. The tertiary objective assembly is corrected for a water/coverslip/water medium (not water/ coverslip/air). It can perform well if operated exactly at the surface of the coverslip, but deviations in alignment that push the image into the coverslip (or out into the air) will produce strong aberrations, so it can be challenging to align and keep stable (and hydrated). In addition, the bulkiness of the coverslip-water assembly requires longer working distance

objectives in the remote refocus to avoid mechanical collision from the tilt. In practice this limits the choice of optics (and tilt range) and can force a reduced numerical aperture on the air objective.

Inspired by OPM and eSPIM, the AMS-AGY v1.0 objective (aka Snouty)[5,6] was developed to eliminate the previous trade-offs and compress the opto-mechanical difficulty into a single dedicated component (Supp. Fig. 5B). The NA 1.0 objective features a monolithic glass tip with zero working distance; this tip is optically equivalent to an oil/coverslip/air interface but alignment-free and mechanically stable. The tip also features an anti-reflection coating to maximize collection from the full hemisphere of rays (as noted previously NA 1.0 in an air collects from the full 90 deg half-angle). The zero working distance is another critical feature; the large refractive index mismatch at the glass-air interface produces strong spherical aberrations that vanish only at this boundary. The high refractive index of the glass tip compresses the collection half-angle so the tip can be shaved off (Supp. Fig. 5) to allow a range of tilt angles from 0-45deg. This excellent mechanical clearance allows image collection as close as 100um from a planar boundary. In practice this means the AMS-AGY objective can be paired with objectives with the highest numerical apertures and therefore the maximum collection efficiency. Infinity and color corrected, this component enables an extensive suite of design options as detailed in the High NA single-objective light-sheet (SOLS) article of 2019.

The Snouty v1.0 objective enabled 'bolt-on' SOLS designs with uncompromised numerical aperture, and is the ideal microscope for many light-sheet applications. However, the design of the v1.0 lens was constrained by mechanical and economic considerations, ultimately limiting the field of view (FOV) to 150um diffraction-limited (250um to the shaved edge, Fig. 5B). The economic argument is obvious: manufacturing difficulty and cost typically increase with field of view, and a costly lens would increase the prototyping risk and lower uptake of the technology. The mechanical limitations are more subtle; for the highest numerical aperture SOLS designs, the air objective that opposes the Snouty lens in the remote refocus can have working distances as short as 200um. So as the Snouty lens is tilted, the field of view (housed in glass) moves towards a collision with the opposing lens i.e. the Snouty FOV competes directly with the working distance of the paired objective in the remote refocus.

To overcome the limits on field of view the Snouty v2.0 prototype (aka KingSnout) was developed with a 3x boost on FOV compared to the v1.0 lens i.e. 450um diffraction limited and 450um to the shaved edge (Supp. Fig. 6&7). The large field was partly achieved by increasing the budget for design and manufacture (it is a larger and more complex lens) but also by eliminating the margin between the diffraction limited FOV and the shaved edge. This enables the lens to be used 'off-axis' in the tightest of spaces without losing optical quality. Snouty v2.0 is also ground at a more aggressive angle (55 deg vs 45 deg, see Supp. Fig. 6A) which combined with the reduced margins gives the maximum clearance for tilting in the remote refocus. It is a

strict upgrade to the v1.0 objective and compatible with all SOLS designs. The discussion of lens specifications brings to the forefront some important considerations that should be emphasized.

The AMS-AGY objectives are exactly as specified: NA 1.0 with fields of 150um (v1.0) and 450um (v2.0), where NA 1.0 actually means the lens will image stigmatically at NA 1.0, not that it merely collects at this NA. How these specifications translate into the object space and the resulting volumetric imaging performance is subtle and beyond the scope of this section. However it should be noted that these objectives can be used beyond the specified fields, by up to a factor of approximately 1.7x, but not at NA 1.0. So a less genuine, but perhaps more typical specification would be NA 1.0 with fields of 250 um (v1.0) and 750 um (v2.0) and is something to bear in mind when considering a SOLS design.

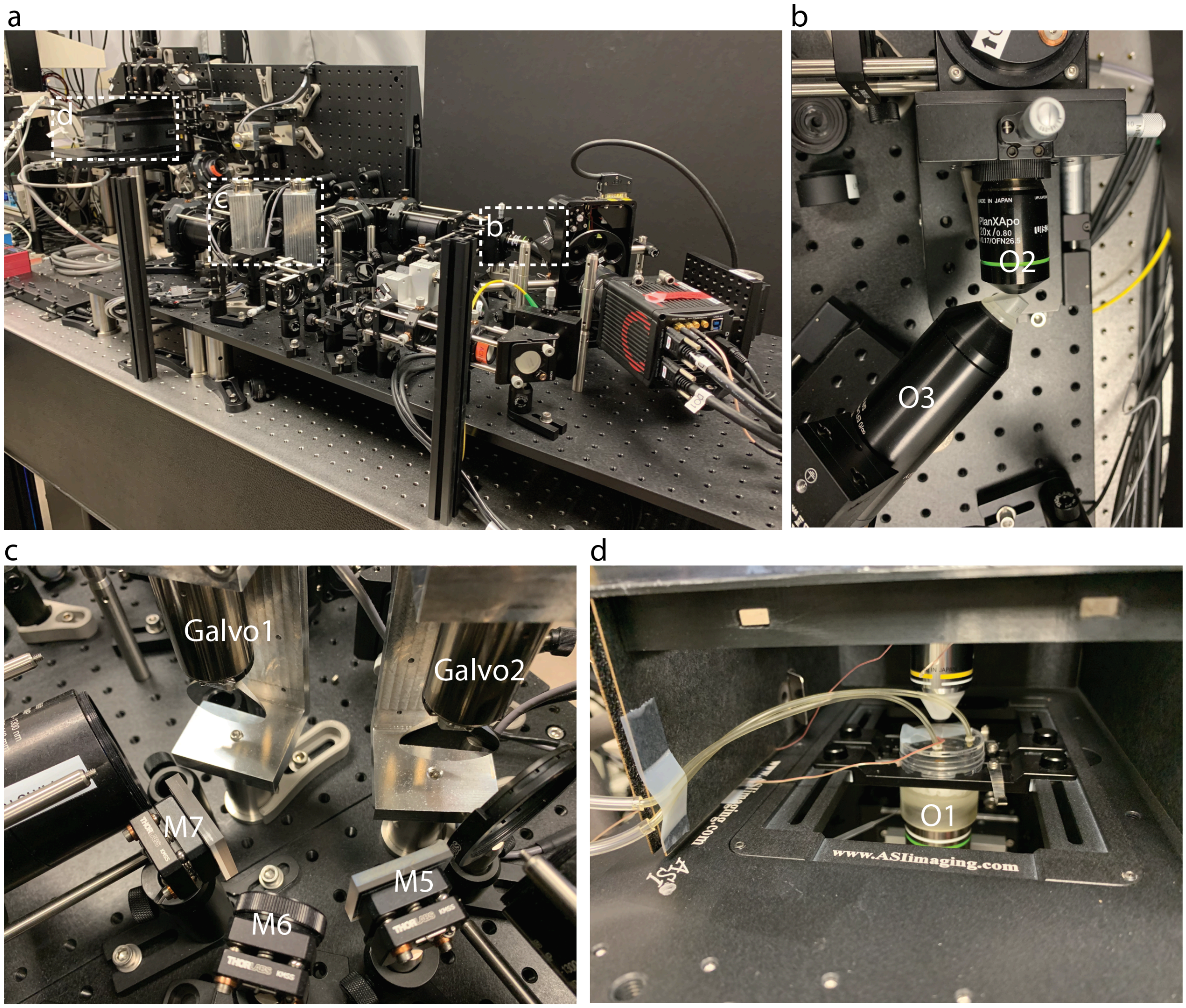

Supplementary Figure 1. Photos of the optical setup of the microscope. (a) Global view of the setup. (b-d) shows highlights some of the key parts of the microscope. (b) Remote focusing objective assembly, O2 and O3. (c) Dual view switching module. (d) Sample stage and environmental chamber.

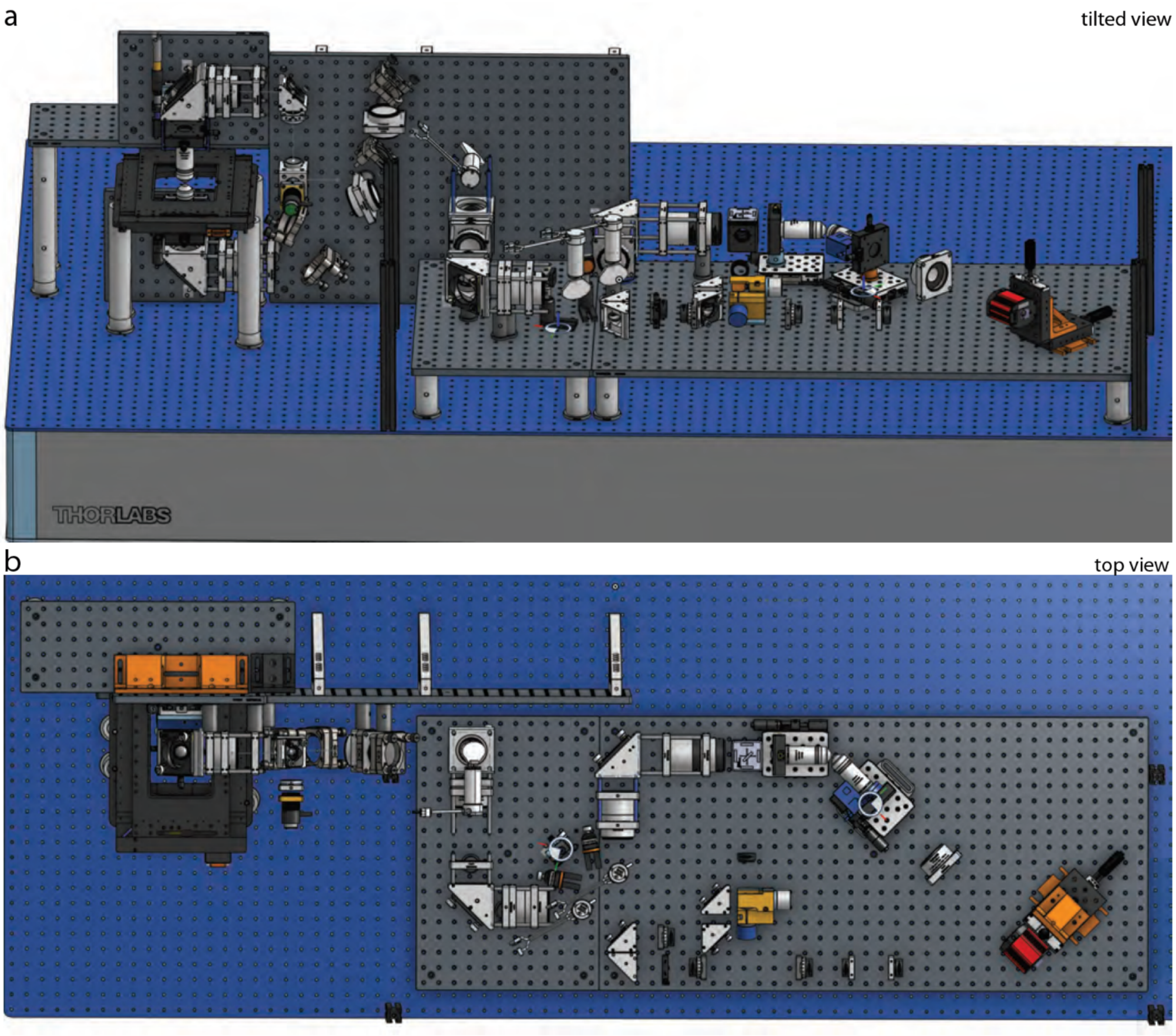

Supplementary Figure 2. 3D rendering of the optical setup of the microscope. The setup is shown from two different views.

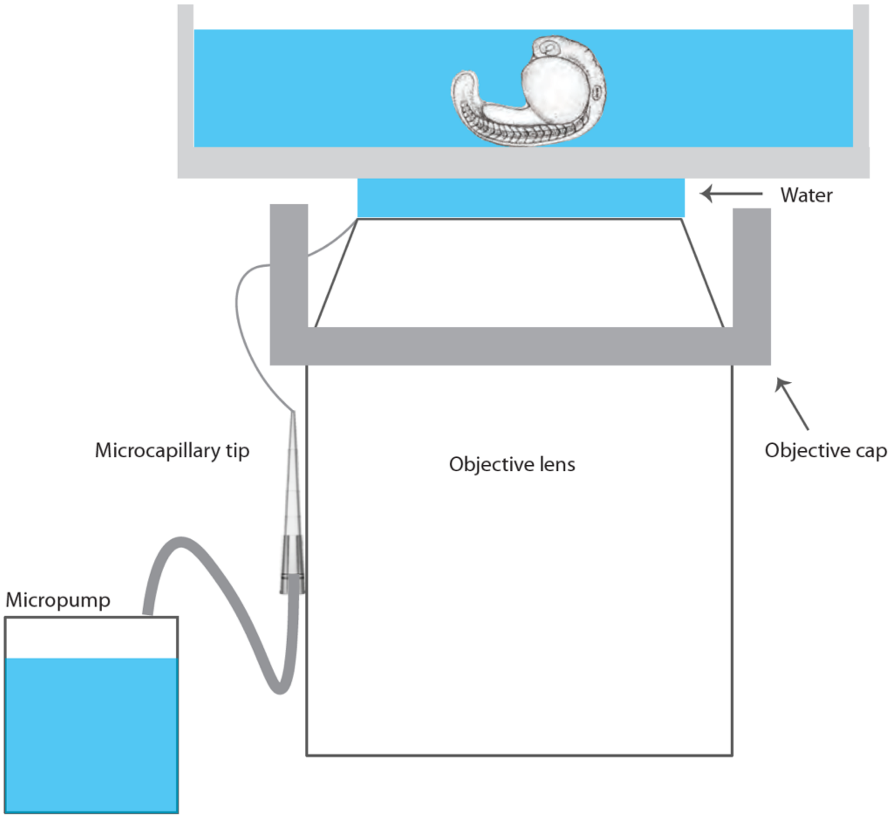

Supplementary Figure 3. Water dispenser for water immersion lens. For long-term imaging, we built a water dispenser to automatically supply immersion water between the primary objective and the sample. A micropump (part of a LeicaWater Immersion Micro Dispenser) pumps water through a microcapillary tip (Eppendorf Microloader) to supply the immersion water to the primary objective. A custom-designed objective cap is 3D-printed using an elastic resin (Elastic 50A, Formlabs). The cap on the objective serves as water reservoir in case of excessive water supply from the pump. The microcapillary tip is glued on the cap for support. The micropump is controlled via serial command to supply water at desired time intervals during imaging.

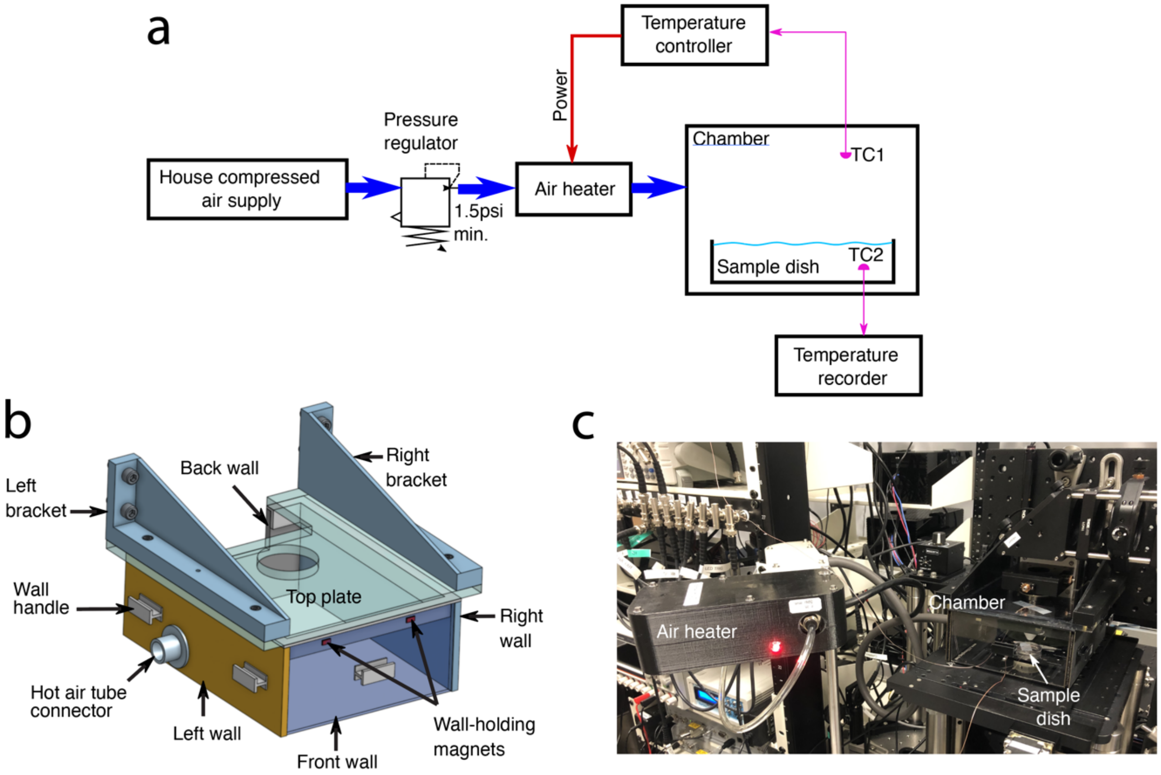

Supplementary Figure 4. Environmental chamber. (A) Diagram of the temperature control system for the environmental chamber. The air heater was custom-built around an Omega Engineering AHP-3741 "resistive air process heater", plus an air pressure switch (McMaster-Carr 40925K92) and two thermal fuses (Cantherm T22A13005DFFBG0E, and L5016724DELB0XE) added for safety. The temperature controller is an Omega Engineering CS8DPT PID desktop controller. TC1 and TC2 are K-type thermocouples used to control the air temperature, and record the sample water temperature, respectively. (B) Main components of the environmental chamber. (C) Actual setup of the environmental chamber and its air heater on the microscope. For imaging of zebrafish embryo development, the sample temperature is kept at 28°C.

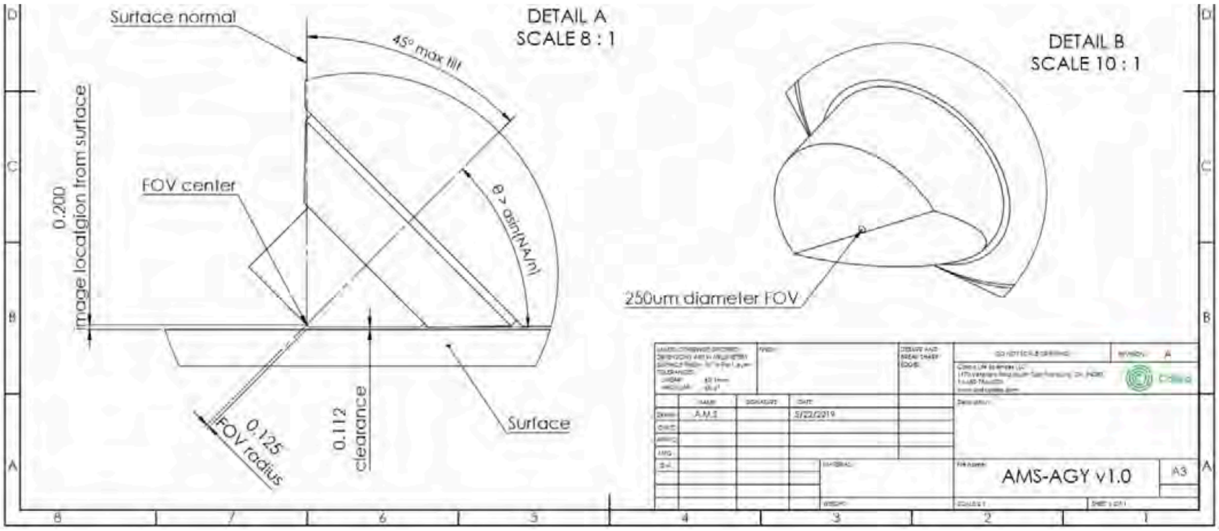

Supplementary Figure 5. Technical drawing for AMS-AGY V1.0 objective.

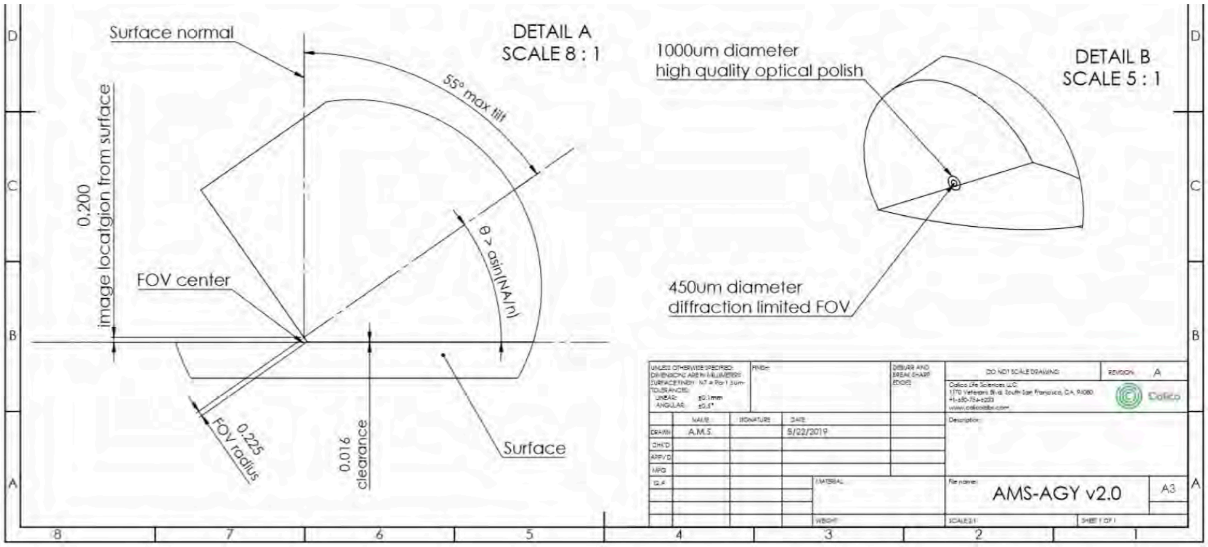

Supplementary Figure 6. Technical drawing for AMS-AGY V2.0 objective.

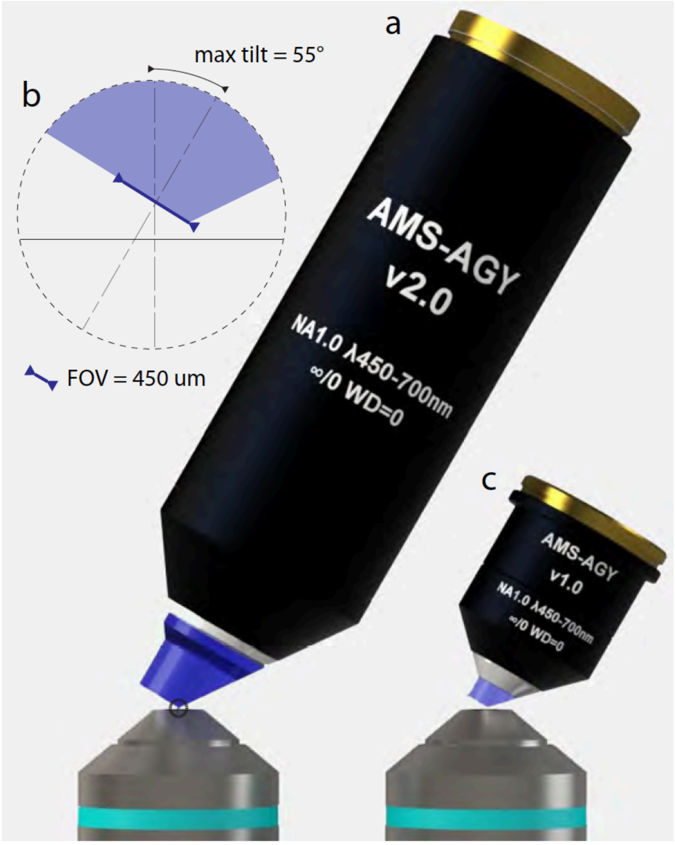

Supplementary Figure 7. Comparison of the AMS-AGY v1.0 and AMS-AGY v2.0 objectives.

Supplementary Table 1. Comparison of scanning modalities in Oblique Plane Microscopy.

Scanning mode		Range	Speed	Motion blur	Peak laser intensity	Image quality across scan range	Stitching artefacts
Galvo scanning		Small	Very fast	No	Normal	Variations[1]	No
Galvo scanning, with stitching		Large	Fast[2]	No	Normal	Variations	Yes
Stage scanning	Stepwise	Large	Very slow	No	Normal	Consistent	No
	Continuous	Large	Fast	Yes	Normal	Consistent	No
	Continuous, With small scanning step	Large	Slow	Minimized	Normal	Consistent	No
	Continuous, With laser strobing	Large	Fast	Minimized	High	Consistent	No
	LS3	Large	Fast[2]	No	Normal	Consistent	No

1. With galvo scanning, the imaging quality varies cross the scan range because the illuminated plane is moving with respect to the optical axis. The more this plane moves away from the optical axis, the faster the image quality degrades. With stage scanning, this issue is avoided as the illuminated plane is static with respect to the optical axis, or within a few µm in case of LS3.

2. See Supp. Fig. 8b for speed comparisons between tiled galvo scan and LS3.

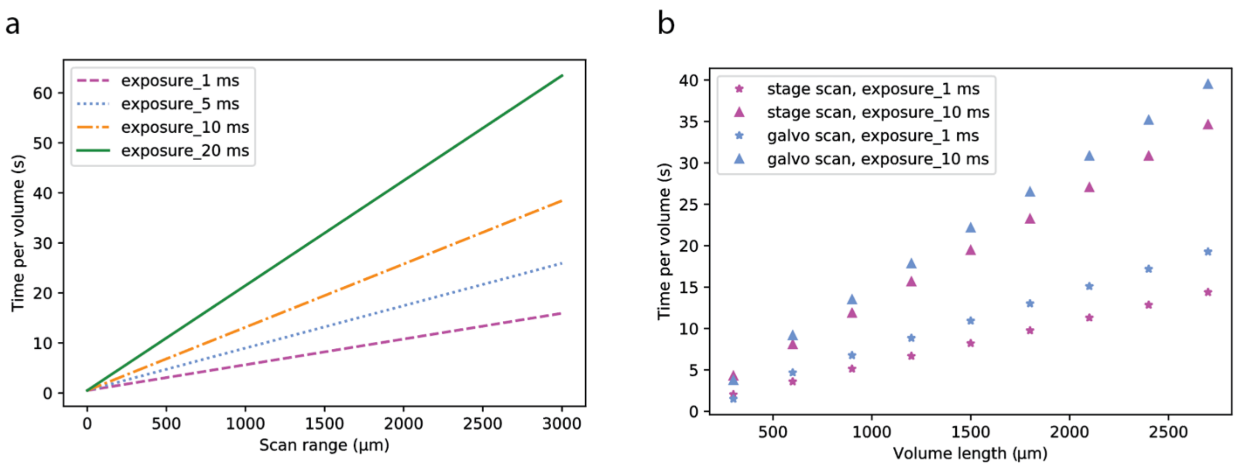

Supplementary Figure 8. Simulation of the temporal resolution of the microscope comparing tiled galvo scanning versus LS^3 scanning. (a) The time required to acquire a 3D dataset for LS^3 scanning as a function of the scan range is: $T_{volume} = n_{frames} * t_{acquistion} + t_{flyback} + t_{additional}$, where T_{volume} is the time per volume, n_{frames} is the number of frames (2D images) within the 3D volume, $t_{acquistion}$ is the acquisition time per frame, $t_{flyback}$ is the time for the stage to go back to the initial position (or next position in case of multiple position imaging) after each scan. Moreover, $t_{additional}$ is the additional time needed for stage movement including the time required for software communication between computer and stage, for the stage to settle mechanically, and for the stage to accelerate and deaccelerate. Finally, n_{frames} is equal to the scan range divided by the scan step and $t_{acquistion}$ is the exposure time plus the readout time of the camera. (b) Imaging time comparison of LS^3 scanning versus tiled galvo scanning as a function of the length of the volume. Imaging time for tiled galvo stage takes into consideration of both the time required to acquire each tile (corresponds to a 300 µm galvo scan), and the time needed to move the stage between tiles and after the last tile. When the volume length to be imaged is 300 µm (or less), no tiling is needed, and galvo scan is faster. However, when the volume to be imaged is larger than 300 µm along the main axis, tiled scan is required and leads to slower imaging than LS^3 scanning. This is mostly due to the multiple movements of the stage between consecutive tiles and after the last tile to go back to the starting position. Note LS^3 is as fast as continuous stage scanning but does not suffer from its drawbacks such as motion blur, photodamage or bleaching (when used in conjunction with light-sheet strobing to reduce motion-blur). Other key parameters for the simulation are: the scan step is set to 1.2 µm; The readout time per frame is 5 ms (for a region of interest of 1024 * 2048 pixels on the camera); The maximum scan speed of the stage is 7 mm/s; $t_{additional}$ is set to 500 ms, a reasonable number considering that the acceleration and deacceleration time is ~ 100 ms, and the settling time is also on the order of 100 ms.

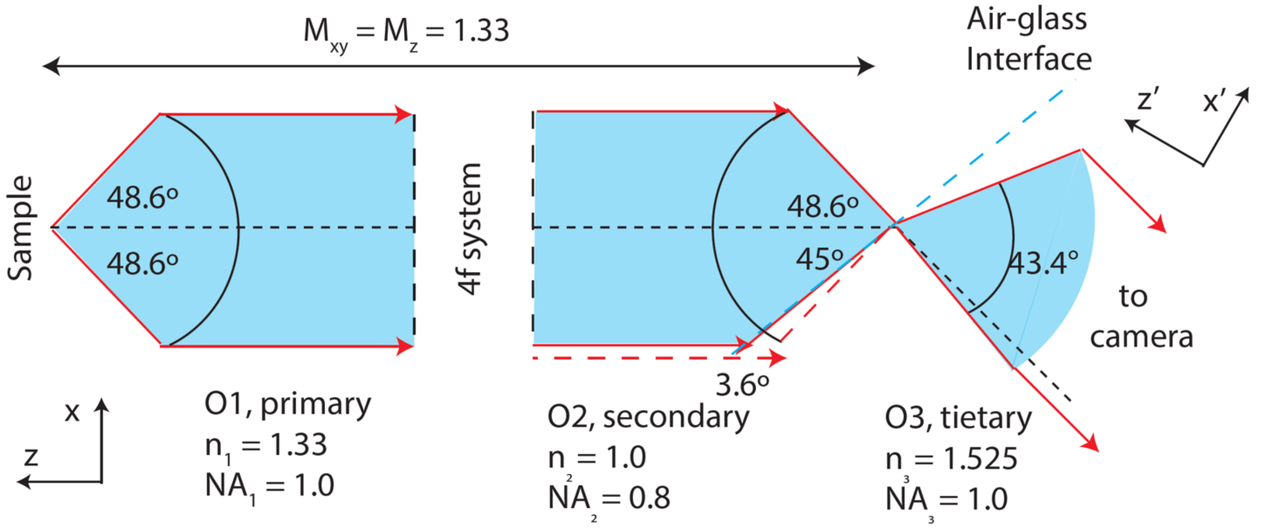

Supplementary Figure 9. Diagram illustrating close to full NA detection along the x' axis. Blue shades indicate the light cone collected by each objective; red lines represent the light rays with the highest incident angle; black dashed lines show the optical axis. The intermediate image at the focal space of O2 is magnified by 1.33 (equal to NA1/NA2) along both the lateral and axial directions to minimize the aberration of the intermediate image. The blue dashed line indicates the position of the air–glass interface. All the light passing though O2 is refracted at this interface and enters O3, except for only a small portion of the light being cropped by the interface (left side between the blue and red dashed lines). Along the x-axis, the NA could be estimated by 1.33*sin((45° + 48.6°)/2) = 0.97. Normal to the tilt direction (y-axis), no clipping happens at the air-glass interface. Thus an theoretical NA of 1.0 can be used.

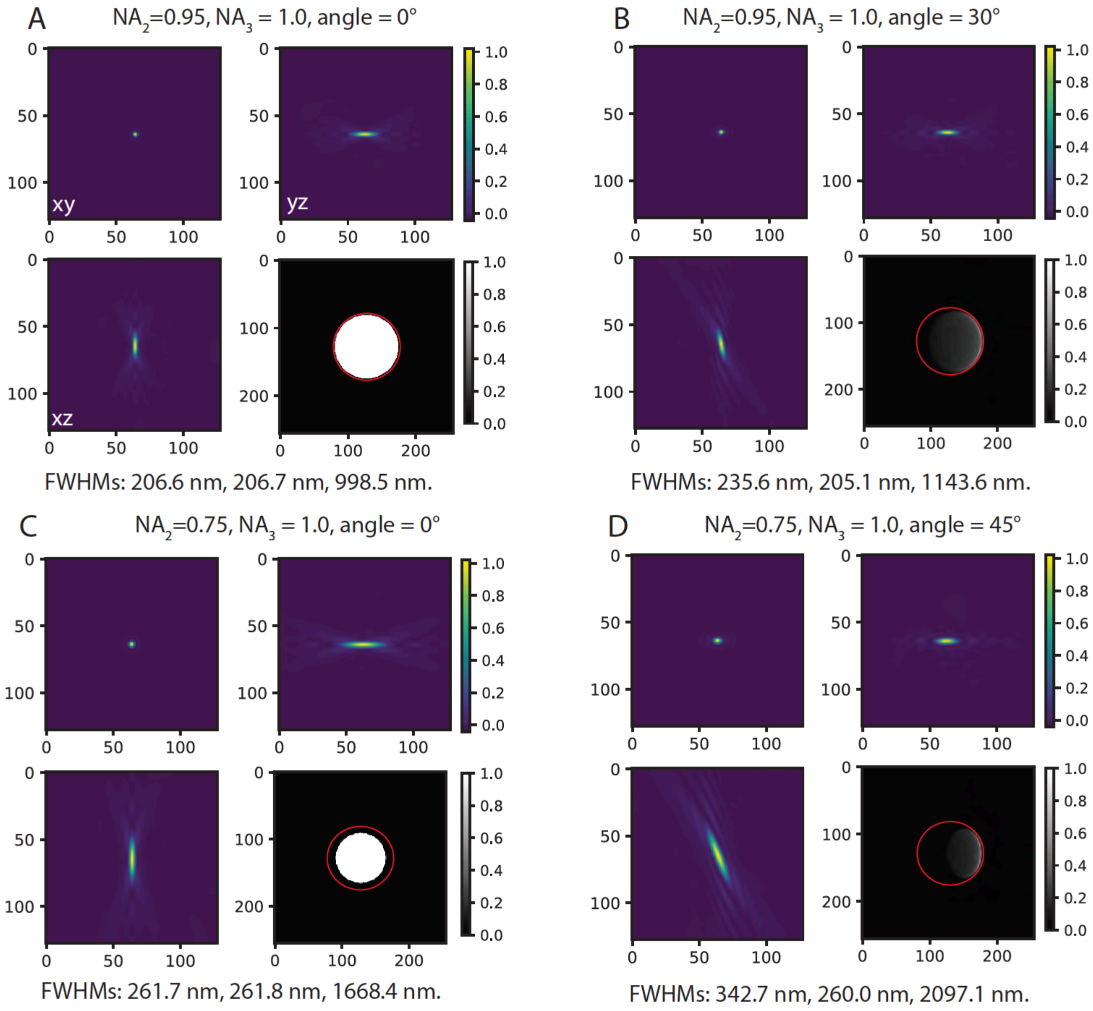

Supplementary Figure 10. Simulated PSF and pupil function of an eSPIM or DaXi system. The system is simplified to only containing the secondary (O2) and tertiary (O3) objectives. A plane wave is assumed to pass through O2 and the pupil function is estimated after the light exits O3. The 3D PSF is then simulated from the pupil function. O2 (NA 0.75 or 0.9) and O3 (NA 1.0) are respectively air and solid immersion objectives. The two objectives are either along a straight line (i.e., angle = 0) or orientated by an angle (30° or 45°) between their optical axes. (a) and (b) show the simulated PSF (xy, xz and yz cross sections are shown) and pupil function (gray images) for the high NA situation, like that reported in 1. The red circles indicate the extend of the pupil of O3. (c) and (d) show the simulated PSF and pupil function for the optical system presented in this paper. When the angle between the two objectives is not 0_, the effective pupil function of the imaging system shows a compression of the light towards one dimension. The resulting PSF is therefore not straight along the z axis, but has an angle to it, which is 10° for (b) and 20° for (d). The PSFs are fitted by a 1D Gaussian Function along the three principal axes to obtain the FWHMs, given at the bottom of the images. The tilted PSFs (b and d) are first rotated to have their principal axes along the x-, y- and z-axes and then fitted with 1D Gaussian Function to get the FWHMs. The FWHMs along y are comparable with O2 and O3 either along a straight line or tilted, suggesting that the effective NA is the same as the secondary objective. The FWHMs along the two other directions are

slightly wider when the two objectives are tilted due to the asymmetry of the pupil function. Note that the FWHMs are adjusted by 1.33 to reflex the magnification from the primary objective (water) to the secondary objective (air).

Supplementary Table 2. FWHM measurements of fluorescence beads at various imaging parameters.

Excitation wavelength (nm)	Figure	Magni-fication	Pixel size (nm)	Field of view (um) width* depth	FWHM_x'' (nm) (mean +- std)	FWHM_y (nm) (mean +- std)	FWHM_z'' (nm) (mean +- std)
488	Fig1b	44.3	147	150*106	479.87 +- 28.04, n = 156	379.22 +- 20.94, n = 156	1864.88 +- 174.30, n = 156
561	NA	44.3	147	150*106	573.38 +- 60.64 nm, n = 48	479.78 +- 54.25 nm, n = 48	2385.36 +- 290.96 nm, n = 48
639	NA	44.3	147	150*106	631.74 +- 46.75 nm, n = 67	540.64 +- 31.08 nm, n = 67	2678.85 +- 212.50 nm, n = 67
488	Sup Fig16a	29.6	220	451*319	576.30 +- 57.43 nm, n = 529	439.65 +- 52.23 nm, n = 529	2316.95 +- 346.82 nm, n = 529
561	Sup Fig16b	29.6	220	451*319	625.64 +- 76.25 nm, n = 439	523.31 +- 73.33 nm, n = 439	2502.99 +- 334.72 nm, n = 439
639	Sup Fig16c	29.6	220	451*319	741.87 +- 82.76 nm, n = 479	640.55 +- 67.00 nm, n = 479	3241.50 +- 475.81 nm, n = 479
488	Sup Fig16d	14.8	440	901*319	857.98 +- 126.45 nm, n = 367	726.16 +- 112.32 nm, n = 367	3014.62 +- 600.13 nm, n = 367
561	Sup Fig16e	14.8	440	901*319	895.29 +- 97.33 nm, n = 398	729.93 +- 73.67 nm, n = 398	3192.92 +- 541.60 nm, n = 398
639	Sup Fig16f	14.8	440	901*319	1068.56 +- 137.33 nm, n = 367	1177.29 +- 82.57 nm, n = 367	4011.22 +- 688.23 nm, n = 367

Supplementary Table 3. FWHM measurements of fluorescence beads, imaged using stage scanning with and without light sheet stabilization.

With LS3	Scanning step (nm)	FWHM_x'' (nm) (mean +- std)	FWHM_y (nm) (mean +- std)	FWHM_z'' (nm) (mean +- std)
Yes	200	477.78 +- 35.31 nm, n = 71	372.82 +- 22.32 nm, n = 71	1877.68 +- 204.78 nm, n = 71
	1000	**526.97 +- 55.89 nm, n = 56**	404.22 +- 43.27 nm, n = 56	1869.52 +- 280.90 nm, n = 56
No	200	499.16 +- 36.80 nm, n = 76	376.77 +- 31.33 nm, n = 76	1896.79 +- 233.94 nm, n = 76
	1000	**931.97 +- 71.24 nm, n = 18**	410.56 +- 42.46 nm, n = 18	2060.99 +- 304.51 nm, n = 18

Note: The FWHM_x'' is about 2 times when the scanning step is at 1000 nm compared to that of 200 nm when the light sheet stabilization is off, indicating the presence of motion blur due to stage scanning.

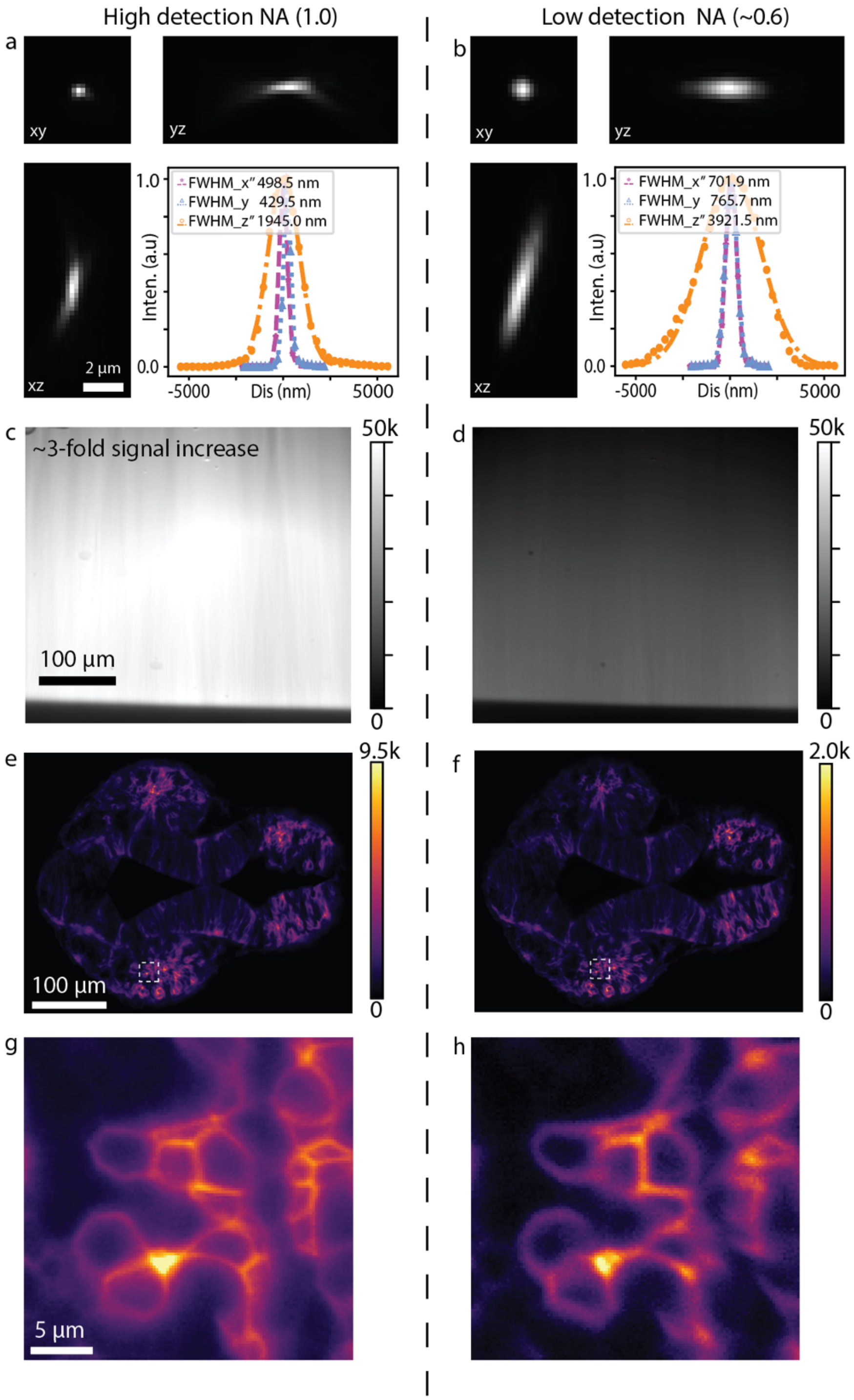

Supplementary Figure 11. Comparing the resolution and signal of high and low detection NA light sheet systems. To demonstrate the advantage of high NA detection with our microscope, we inserted an iris right after O3. By closing the iris, we reduced the effective detection NA to ~ 0.6. (a) and (b) show representative PSFs measured with 100 nm green fluorescence beads. The resolution of a low NA system is clearly worse – as expected. (c) and (d) show images of a uniform fluorescein solution under the light sheet illumination. The high NA system collect ~ as 3 time more fluorescence signal which is crucial to image dim samples. (e) and (f) show hindbrain images of a zebrafish embryo (~ 24hpf,

membrane labelled with ef1a-MyrTdTomato[7]). XY slices across 3D volumes are shown. Again, the signal is more than 3 times less with the low NA system. The areas highlighted by the white dashed rectangles are shown in (g) and (h), which clearly demonstrate enhanced higher spatial resolution and signal noise ratio with the high NA system.

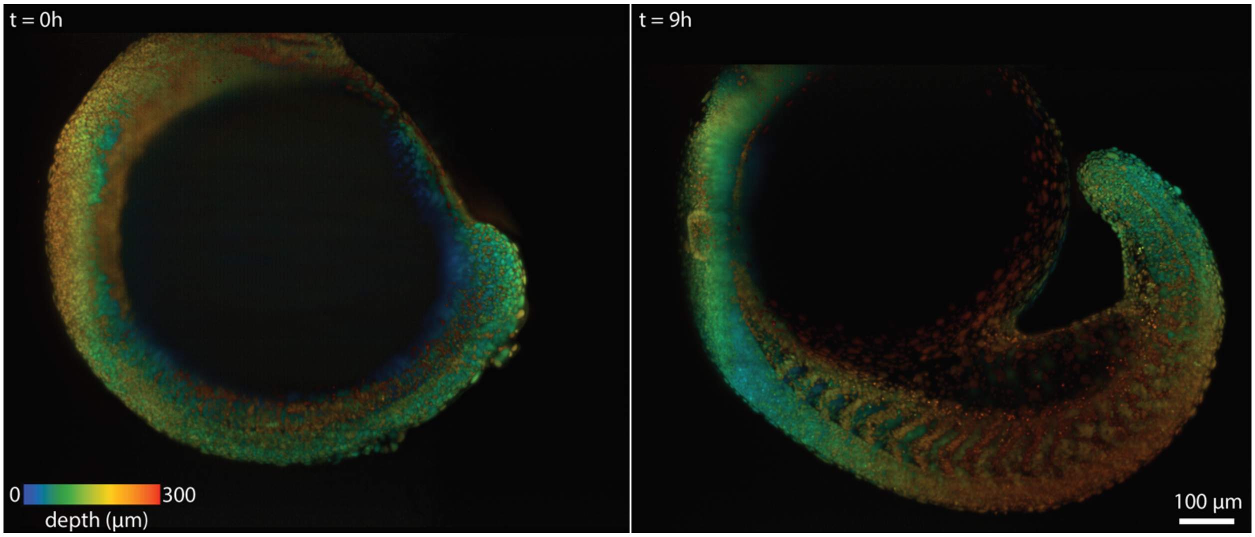

Supplementary Figure 12. Volumetric imaging of zebrafish embryo development. Imaging volume is 1200 µm * 800 µm * 300 µm acquired every 30 seconds (two views) for 9 hours. The images shown are max-intensity projection where the depth is color-coded (blue (red) means close to (away from) the surface). See also Supplementary Movie 3.

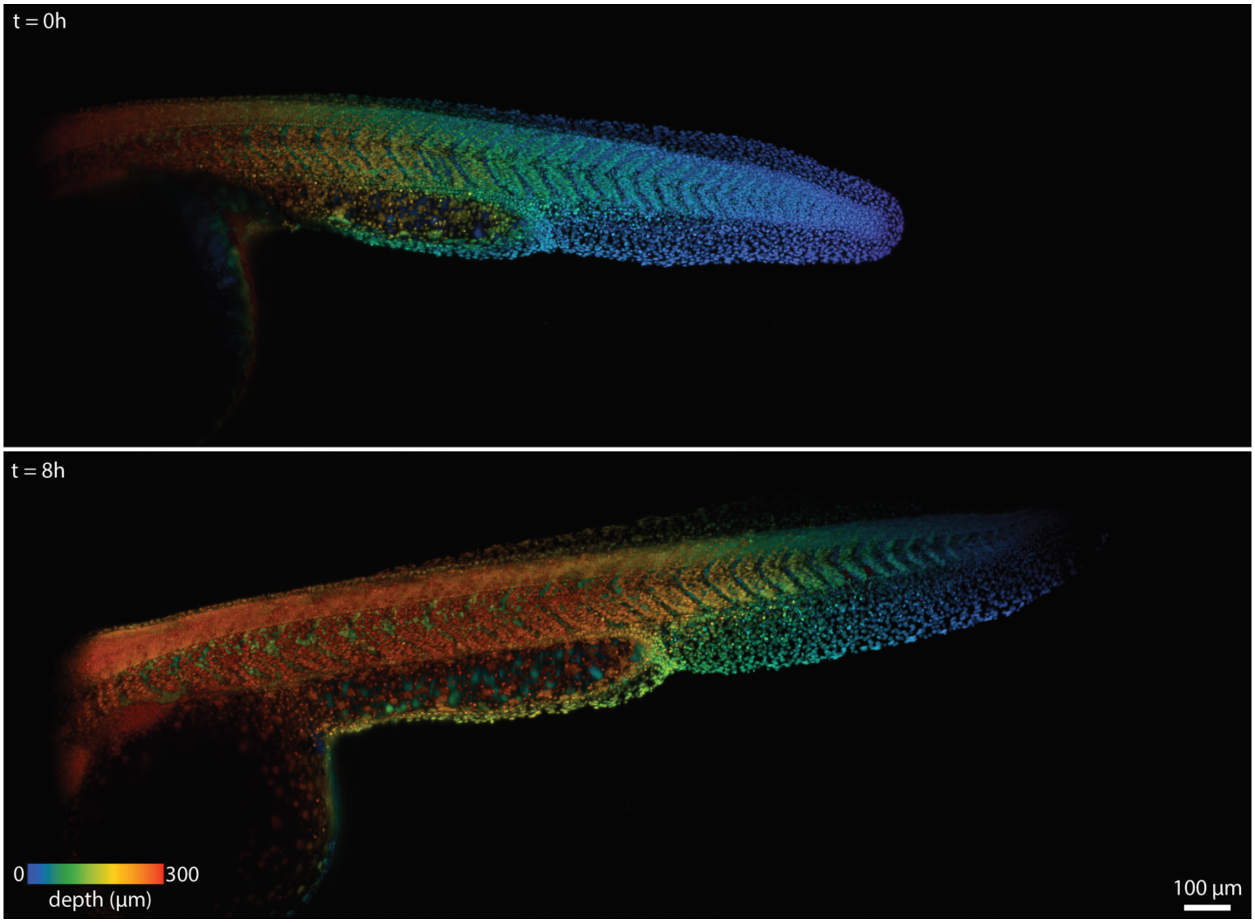

Supplementary Figure 13. Volumetric imaging of zebrafish tail development. Imaging volume is 2200 µm * 800 µm * 300 µm acquired every 73 seconds (two views) for 8 hours. The images shown are max-intensity projection where the depth is color-coded (blue (red) means close to (away from) the surface). See also Supplementary Movie 4.

Supplementary Table 4. Comparison of some key system parameters among state-of-art Oblique Plane Microscopy methods.

**Method**	**eSPIM**	**Snoutscope**	**SCAPE 2.0**	**SoPi**	**dOPM**	**DaXi (this paper)**
Reference	Nature Methods 2019[4]	GitHub 2019[5] Elife 2020[6]	Nature Methods 2019[8]	OE 2018[9], OL 2019[10]	BOE 2020[11]	
Primary objective	Water-immersion 60x 1.27 NA	Silicone-immersion 100x 1.35 NA	Water-dipping 20x 1.0 NA	Water-dipping 20x 1.0 NA	Water-immersion 40x 1.15 NA	Water-dipping 20x 1.0 NA
Microscope Geometry	Inverted	Inverted	Upright	Upright	Inverted	Inverted
Number of detection views	1	1	1	1	2	2
Cumulative theoretical detection NA[1] (tilting axis, x)	1.18	1.28	0.35	0.34	0.58	0.97
Cumulative theoretical detection NA (y-axis)	1.27	1.35	0.60	0.60	0.93	1.0
Field of view (y * z) (width * depth)	70 µm * 20 µm	220 µm * 60 µm	1100 µm * 385 µm	950 µm * 400 µm	295 µm * 209 µm	800 µm * 300 µm
Modified Etendue[2] (µm^2)	2098	22809	88935	79800	33257	232800
Galvo scanning range	100 µm	180 µm	700 µm	500 µm	N.A.	300 µm
Stage scanning mode ()	N.A.	Continuous, small step (100 nm or 200 nm) per frame, galvo mirror static	Continuous, galvo mirror static	N.A.	Using linear actuator, scan range 300 µm	Light Sheet Stabilized Stage scanning (LS3)

1. The Cumulative theoretical detection NA is calculated based on the collection angle of the light cone in the primary objective space (see Suppl. Fig. 9). For eSPIM, it is 1.33*sin((60° + 64.2°)/2)=1.18. For Snoutscope, it is 1.4*sin((60° + 71.8°)/2) = 1.28. For DaXi, it is 1.33*sin((45° + 48.6°)/2)=0.97.

2. The modified etendue is defined as the width of the field of view (y-axis) * the depth of the field of view (z-axis) * detection NA (x-axis) * detection NA (y-axis).

The modified etendue here gives the theoretical upper bound of the optical throughput of those methods independently of particular implementations such as choice of camera.

Practical comparison among those methods would depend on the particular implementations of each method, for example an estimation of the effective spatial resolution across the whole imaging volume, which is not available for many of the methods.

In our current system, the full field of view is sampled at 440 nm due to limited number of pixel of the camera (2048*2048). Using a camera with smaller pixel size and more pixels would increase the practical throughput of our system.

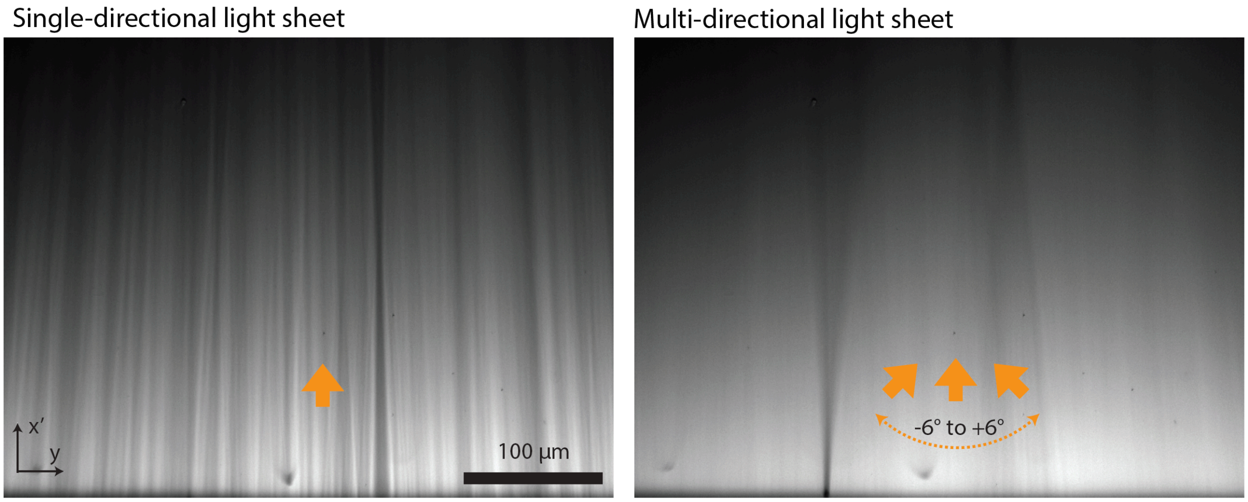

Supplementary Figure 14. Stripe reduction with multi-directional light sheet[12]. Images of fluorescein water under oblique light sheet illumination are shown. When illuminated with a single-directional light sheet, one can clearly see some stripes due to sample absorption and obstruction of the illumination light. By contrast, the stripe artefacts are greatly reduced when the sample is exposure with a multi-directional light sheet. The direction of the light sheet is controlled by the two-axes galvo (Extended Data Fig. 1a) so that the light sheet sweeps continuous from -6° to 6° within the illumination plane during the acquisition. Orange arrow indicates the light propagation direction.

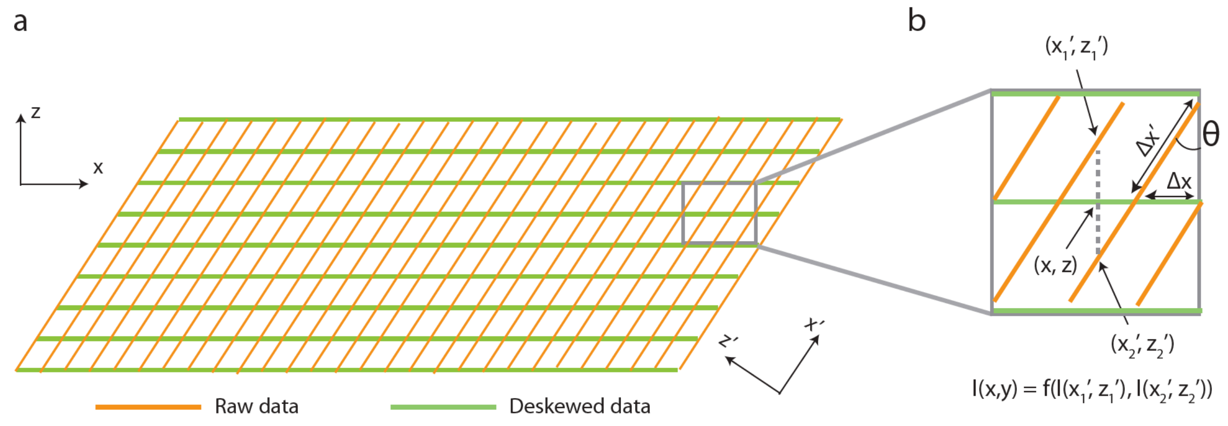

Supplementary Figure 15. Resampling of data to sample coordinates. The 3D stack is initially in the x'yz' coordinates. To convert the stack to sample (i.e. xyz) coordinates, the intensity of each voxel in the xyz coordinates is the result of interpolation between two neighboring voxels (in the x'yz' coordinates) along the vertical direction. The scanning distance between two adjacent planes in the raw data is set experimentally such that Δx_0 is always an integer value of the pixel size long the x'-axis so that no interpolation is needed within along the x'-axis.

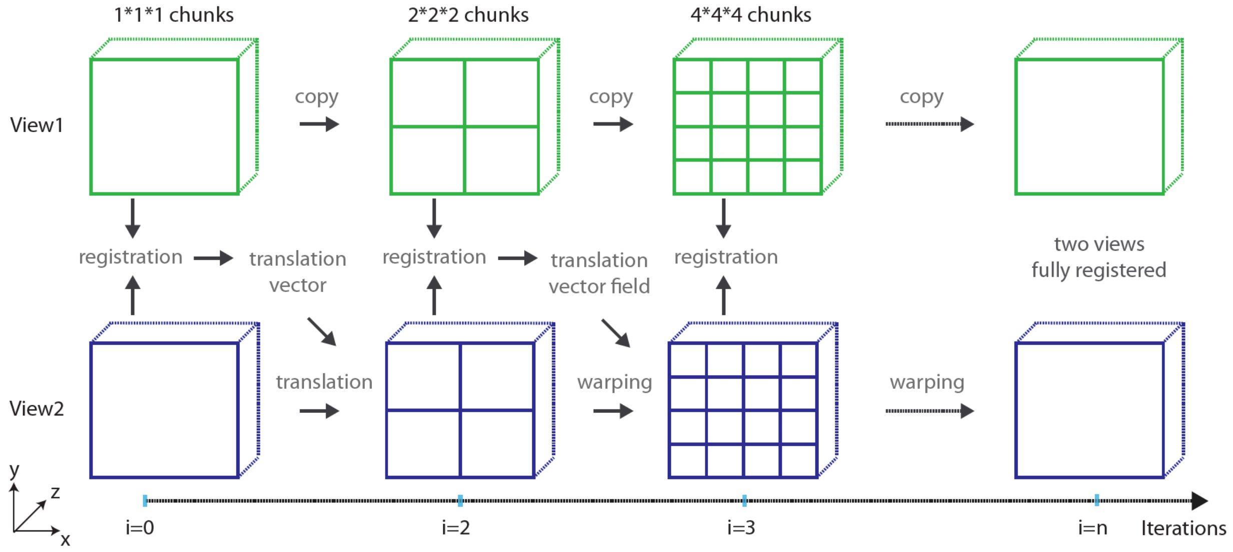

Supplementary Figure 16. Warp registration. The image from the second view is registered with respective to that of the first view using an iterative multi-scale wrap approach. Within each iteration: the images are divided into chunks along all three axes by a factor of 2^i where i is the current iteration number; corresponding chunks from both views are registered separately with a translation model to produce a translation vector; a vector field is then calculated based on all the translation vectors; the image of the second view is warped according to the vector field. This procedure repeats until the max number of iteration or the minimal size of the chunk is reached. In this work, the max iteration number was set to 4 and the minimal chunk size was set to 32 * 32 * 32.

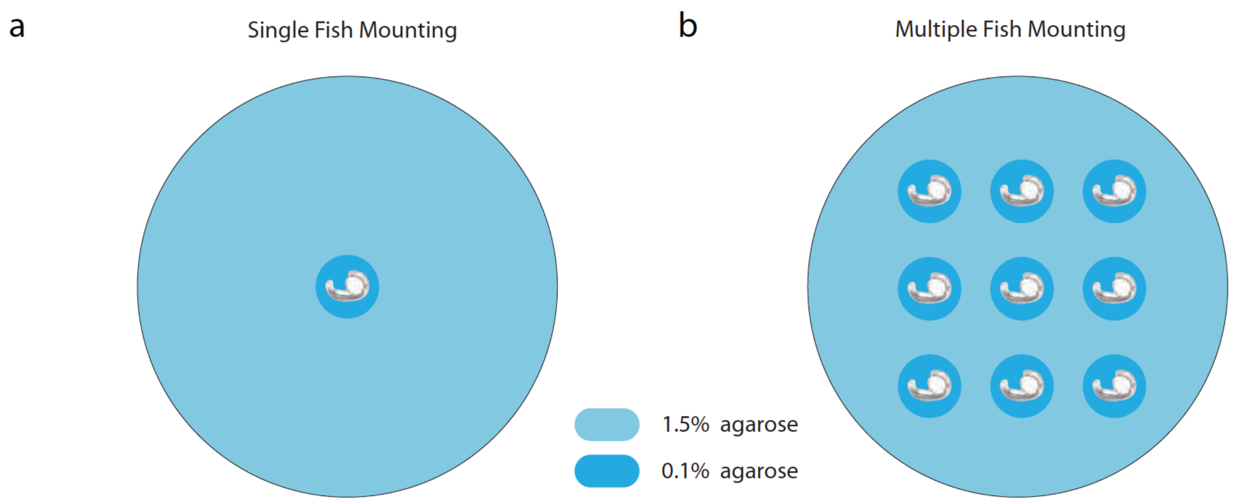

Supplementary Figure 17. Zebrafish embryo mounting. 35mm cell culture dish with glass bottom (TCD35GB20, Stellar Scientific) are firstly filled with 1.5% agarose gel, with one or multiple empty spaces (~ 3mm diameter). The zebrafish embryos are then embedded in 0.1% agarose gel and mounted inside the holes.

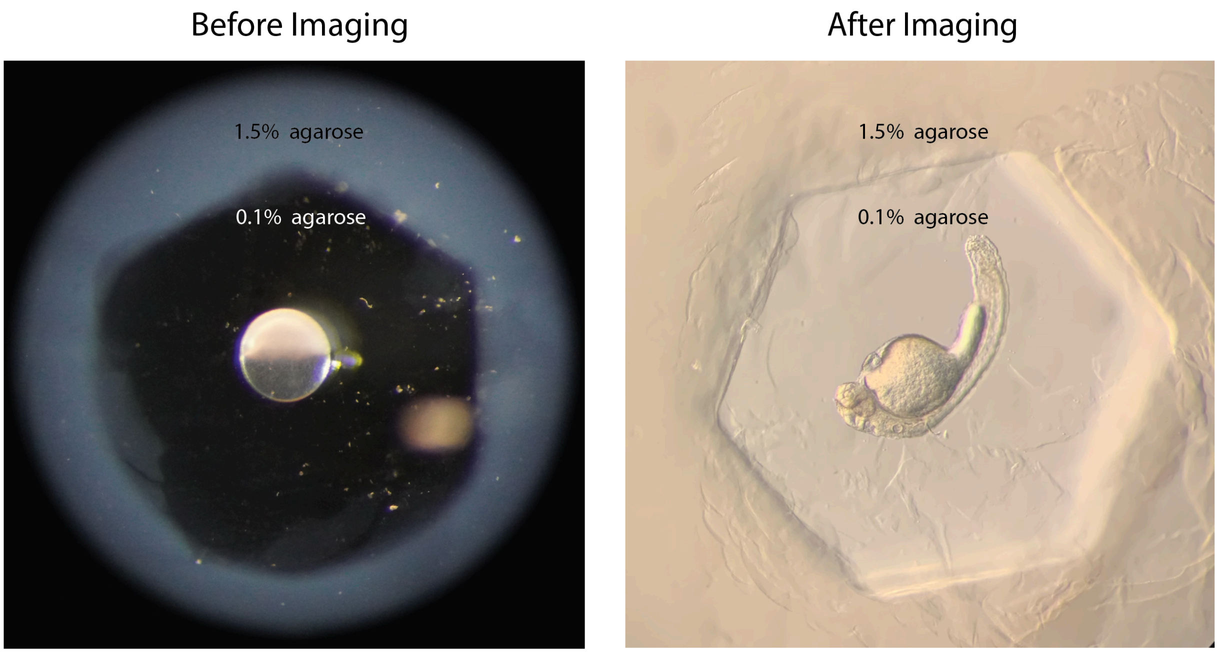

Supplementary Figure 18. Zebrafish embryo mounting. The embryo is mounted in a 35 mm petri dish. Photos are taken before and after imaging.

Supplementary Table 5. List of materials used to construct the DaXi microscope.

Name of Material/ Equipment	Description	Catalog Number Company	Catalog Number
**Optics shown in Figure**			
O1	1.0NA 20x water objective	Olympus	XLUMPLFLN 20XW
O2	0.80NA 20x air objective	Olympus	UPLXAPO20X
O3	1.0NA 20x Solid Immersion	Special Optics	Calico AMS 2.0
Scanning and swtiching galvos	20 mm Galvo mirror, 40 Degree Optical Angle, S4 mirror	Cambridge Instrument	6SD12205
2-axes galvo	galvo to adjust incident angles of the light sheet	Cambridge Instrument	6SD12056
Emission Filter	Single band filter	Chroma	ET525/50m
Emission Filter	Single band filter	Chroma	ET610/75m
Excitation Filter	Quad band filter	Chroma	89402x
Dichroic mirror	Quad band filter	Chroma	ZT405/488/561/640rpcv2-UF3
TL1-TL3	Super wide tube lens	Olympus	SWTLU-C
TL4-TL6 components	Laser Scanning Tube Lens	Thorlabs	TTL200MP
TL4-TL6 components	2 inch achromatic lens	Thorlabs	AC508-750
TL4-TL6 components	2 inch achromatic lens	Thorlabs	AC508-500
TL4-TL6 components	2 inch achromatic lens	Thorlabs	AC508-750-A-ML
TL7_alternative	Tube Lens, f = 200 mm,	Thorlabs	TTL200
TL7_alternative	Tube Lens, f = 165 mm,	Thorlabs	TTL165A
TL7_alternative	Tube Lens, f = 100 mm	Thorlabs	TTL100A
CL1	cylindrical lens 50 mm	Thorlabs	LJ1695RM-A
CL2 and CL3	cylindrical lens 200 mm	Thorlabs	LJ1653RM-A
Fiber output	Protected Silver Reflective Collimator, 450 nm - 20 µm, Ø2 mm Beam, FC/APC	Thorlabs	RC02APCP01
**Other major devices**			

Piezo for O3	Fast PIFOC® Piezo Nanofocusing Z-Drive, 100µm	Physik Instrumente	PD72Z1SAQ
Laser	Stradus series module at 488nm, 50mW output with ~0.8 mm beam	Vortran	Stradus® 488 – 50
Laser	Stradus series module at 561nm, 50mW output with ~0.7 mm beam	Vortran	Stradus® 561 – 50
Data acquistion card	CompactDAQ chassis (8 slot USB)	National Instrument	cDAQ-9178
Data acquistion card	4 Ch, +/-10 V, 16-Bit, 100 kS/s/ch, AO Module	National Instrument	NI 9263 DSUB
Data acquistion card	8-Channel, 100 ns, TTL Digital Input/Output Module	National Instrument	NI 9401
Sample translation	MS-2000 FLAT-TOP XY AUTOMATED STAGE	ASI	MS-2000
Fiber input	FiberPort, FC/APC	Thorlabs	PAF2-A7A
**Miscellaneous**			
For galvos	HOOK-UP STRND 14AWG Wires for Galvo controller	Digikey	NA
For galvos	SWITCHING POWER SUPPLIES	Astrodyne TDI	MK320S-24
Camera	ORCA-Flash4.0 V3 Digital	Hamamastu	C13440-20CU-KIT
Camera	Interface Kit - Firebird Camlink Board and 2 ea SDR-SDR Caml	Hamamastu	CAMRA-1007-000-KIT
**Custom mounts**			
For galvos	CambrigeGavloMount 2 axes, orthorgonal mount	Protolabs	NA
For O1 mount	ST1XY_PiezoAdapter_bottom	Protolabs	NA
For O1 mount	ST1XY_PiezoAdapter_top	Protolabs	NA
For O1 mount	XR25P_Piezo_adapter_bottom	Protolabs	NA
For O1 mount	XR25P_Piezo_adapter_top	Protolabs	NA
For galvos	20mmGalvoMount - Part 1_v2, custom mount	Protolabs	NA
For O1 water dispenser	Olympus1p00_20x - ObjectiveWaterDispenser	3D-printed	NA
**Thorlabs optomechanics**			

O1-O3 translation	Manual translation stages	Thorlabs	XR25P
O1 translation	Motorized translation stages	Thorlabs	ZFS25B
xy translation	XY Translator with Micrometer Drives	Thorlabs	ST1XY-S
Mirror mount	Right-Angle Kinematic Mirror Mount	Thorlabs	KCB2C
Mirror mount	Right-Angle Kinematic Mirror Mount	Thorlabs	KCB1C
Mirror mount	Polaris® Ø2" Mirror Mount	Thorlabs	POLARIS-K2
Cage system	60 mm Cage Plate, SM2 Threads	Thorlabs	LCP01
Alignment reference	PostMountable<br>Standard Iris, Ø20.0 mm Max Aperture	Thorlabs	IDA20P5
Alignement check	Positive Combined Resolution and Distortion Test Target	Thorlabs	R1L1S1P
Olympus tube lens adpator	Modified SM2-Threaded Mounting Adapter	Thorlabs	SM2AD-M41-SP
Post	Ø1" Pedestal Pillar Post	Thorlabs	RS2P8E
Post	Ø1" Post Holder with Flexure Lock, Pedestal Base	Thorlabs	RSH1.5
Post	Ø1" Pillar Post	Thorlabs	RS1.5
Mirror mount	Compact Kinematic Mirror Mount Hex Adjuster	Thorlabs	KMSS
Translation stage	Compact†25†mm†Travel†Linear†Translation†Stage	Thorlabs	XRN25C
Translation stage	Baseplate†for†Stages†with†2¢†Wide†Dovetails	Thorlabs	XRNB1
Breadboard assembly	Aluminum Breadboard	Thorlabs	MB8
Breadboard assembly	Aluminum Breadboard	Thorlabs	MB1218
Breadboard assembly	Aluminum Breadboard	Thorlabs	MB618
Breadboard assembly	Aluminum Breadboard	Thorlabs	MB1824
Breadboard assembly	Aluminum Breadboard	Thorlabs	MB1836
Breadboard assembly	Aluminum Breadboard	Thorlabs	MB810
Breadboard assembly	Right≠Angle†Mounting†Plate	Thorlabs	AP90
Breadboard assembly	Large Right-Angle Mounting Plate	Thorlabs	AP90L
Breadboard assembly	18" Vertical Bracket for Breadboards	Thorlabs	VB01B
Dichroic mount	Kinematic†Fluorescence†Filter†Cube¨	Thorlabs	DFM1L

Supplementary Table 6. Imaging conditions for all experiments.

Figure	Sample	Fluorescent Label	Excitation (nm)	Imaging Volume (scan range * width * depth * nb views	Voxel Size (x * y * z)	Data processing	Exposure Time (per slice) (per tp) (# tp)
Fig. 3a	zebrafish	h2afva.mCherry	561	3000 µm * 800 µm * 300 µm * 2 views	0.440 µm * 0.440 µm * 1.806 µm	Deskew, Dehazing, Fusion	15 ms 52 s 1 tp
Movie 1	zebrafish	h2afva.mCherry	561	3000 µm * 800 µm * 300 µm * 2 views	0.440 µm * 0.440 µm * 1.806 µm	Deskew, Dehazing, Fusion, Deconvolution	15 ms 52 s 1 tp
Fig. 3c, Movie 2	fly	UAS-NLS-GFP	488	3000 µm * 800 µm * 300 µm * 1 view	0.440 µm * 0.440 µm * 1.204 µm	Deskew	10 ms 27 s 342 tp
Fig. 4	zebrafish	h2afva.mCherry	561	1064 µm * 532 µm * 287 µm * 2 views	0.266 µm * 0.266 µm * 1.880 µm	Deskew, Dehazing, Fusion	30 ms 40 s 100 tp
Fig. 5, Movie 6	zebrafish	h2afva.mCherry	561	1000 µm * 800 µm * 300 µm * 2 views	0.440 µm * 0.440 µm * 1.806 µm	Deskew, Dehazing, Fusion, Temporal stabilization	15ms 18.6 s / fish (167.5 s / 9 fishes) 100 tp
Supp. Fig. 11e-h	zebrafish	ef1a-MyrTdTomato	561	600 µm * 450 µm * 300 µm * 1 view	0.220 µm * 0.220 µm * 0.620 µm	Deskew	40 ms 39 s 1 tp

Extended Data Fig. 7	zebrafish	h2afva.mCherry, DiO cell-labelling solution for membranes	488, 561	3200 µm * 800 µm * 300 µm * 2 views	0.440 µm * 0.440 µm * 1.806 µm	Deskew, Dehazing, Fusion	20 ms 73 s 1 tp
Extended Data Fig. 8a	zebrafish	elav3.GCaMP6f	488	300 µm * 500 µm * 200 µm * 1 view	0.440 µm * 0.440 µm * 8 µm	Deskew	8ms 0.3 s 100tp
Extended Data Fig. 8b	zebrafish	elal3.h2b-GCaMP6f	488	320 µm * 500 µm * 200 µm * 1 view	0.440 µm * 0.440 µm * 7 µm	Deskew	4ms 0.18 s 300 tp
Extended Data Fig. 9	zebrafish	h2afva.mCherry	561	800 µm * 450 µm * 300 µm * 1 view	0.220 µm * 0.220 µm * 0.930 µm	Deskew	40 ms 35 s 1 tp
Supp. Fig. 12 Movie 3	zebrafish	h2afva.mCherry	561	1200 µm * 800 µm * 300 µm * 2 views	0.440 µm * 0.440 µm * 1.806 µm	Deskew, Dehazing, Fusion, Stablization	15 ms 32 s 1079 tp
Supp. Fig. 13 Movie 4	zebrafish	h2afva.mCherry	561	2200 µm * 800 µm * 300 µm * 2 views	0.440 µm * 0.440 µm * 1.806 µm	Deskew, Dehazing, Fusion, Stablization	15 ms 57 s 1 tp

References

1. Wu, Y. *et al.* Spatially isotropic four-dimensional imaging with dual-view plane illumination microscopy. *Nat. Biotechnol.* **31**, 1032–1038 (2013).
2. Chen, B.-C. *et al.* Lattice light-sheet microscopy: Imaging molecules to embryos at high spatiotemporal resolution. *Science* **346**, 1257998 (2014).
3. Dunsby, C. Optically sectioned imaging by oblique plane microscopy. *Opt. Express* **16**, 20306–20316 (2008).
4. Yang, B. *et al.* Epi-illumination SPIM for volumetric imaging with high spatial-temporal resolution. *Nat. Methods* **16**, 501 (2019).
5. Millett-Sikking, A. & York, A. G. High NA single-objective lightsheet. *Github.io* (2019).
6. Sapoznik, E. *et al.* A versatile oblique plane microscope for large-scale and high-resolution imaging of subcellular dynamics. *eLife* **9**, e57681 (2020).
7. Zhao, X. *et al.* Polarized endosome dynamics engage cytoplasmic Par-3 that recruits dynein during asymmetric cell division. *Sci. Adv.* **7**, eabg1244.
8. Voleti, V. *et al.* Real-time volumetric microscopy of in vivo dynamics and large-scale samples with SCAPE 2.0. *Nat. Methods* **16**, 1054–1062 (2019).
9. Kumar, M., Kishore, S., Nasenbeny, J., McLean, D. L. & Kozorovitskiy, Y. Integrated one- and two-photon scanned oblique plane illumination (SOPi) microscopy for rapid volumetric imaging. *Opt. Express* **26**, 13027–13041 (2018).
10. Kumar, M. & Kozorovitskiy, Y. Tilt-invariant scanned oblique plane illumination microscopy for large-scale volumetric imaging. *Opt. Lett.* **44**, 1706–1709 (2019).
11. Sparks, H. *et al.* Dual-view oblique plane microscopy (dOPM). *Biomed. Opt. Express* **11**, 7204–7220 (2020).

12. Huisken, J. & Stainier, D. Y. R. Even fluorescence excitation by multidirectional selective plane illumination microscopy (mSPIM). *Opt. Lett.* **32**, 2608–2610 (2007).
